# Enhanced wind power forecasting using machine learning, deep learning models and ensemble integration

**DOI:** 10.1038/s41598-025-05250-3

**Published:** 2025-07-01

**Authors:** T. A. Rajaperumal, C. Christopher Columbus

**Affiliations:** 1 School of Electrical Engineering, Vellore Institute of Technology, Chennai, Tamil Nadu 600127 India; 2 School of Computer Science and Engineering, Vellore Institute of Technology, Chennai, Tamil Nadu 600127 India

**Keywords:** Energy forecast, Renewable energy, Machine learning, Deep learning, Sustainability, Electrical and electronic engineering, Computational science, Energy science and technology, Engineering

## Abstract

The inherent variability of wind and solar energy introduces fluctuations in power generation, making accurate forecasting essential for maintaining the grid’s stability. This study addresses key research gaps in wind energy forecasting, including the inability of traditional statistical models to capture complex, nonlinear temporal patterns, the underutilization of real-time, location-specific data, the lack of comparative analyses across diverse models and datasets, and the absence of systematic model selection strategies for future forecasting. To overcome these limitations, this study applies advanced machine learning (ML) and deep learning (DL) techniques with systematic hyperparameter tuning to enhance predictive performance. Three scenarios were examined: Case 1 used a Kaggle wind turbine SCADA dataset; Case 2 employed real-time wind data from Aralvaimozhi, Tamil Nadu, India; and Case 3 focused on future forecasting using the best performing models from the earlier cases. A wide range of ML models—Random Forest (RF), Decision Trees, Linear Regression, K-Nearest Neighbors (KNN), Extreme Gradient Boosting (XGBoost), Adaptive Boosting (AdaBoost), and Gradient Boosting—alongside DL models such as Multi-Layer Perceptron (MLP) and Long Short-Term Memory (LSTM) were evaluated. Weather features, particularly wind speed, were incorporated to improve the prediction accuracy. A Stacking Ensemble model was also constructed from the top-performing models to boost robustness and forecast reliability. The performance was evaluated using the Mean Absolute Error (MAE), Mean Squared Error (MSE), Root Mean Squared Error (RMSE), and R-squared (R^2^) metrics. The results showed that Random Forest excelled in Case 1, while Case 2 saw outstanding performance from Random Forest, XGBoost, and the Stacking Ensemble, achieving R^2^ values of 0.995, 0.997, and 0.998; MAE of 0.027, 0.035, and 0.014; MSE of 0.026, 0.014, and 0.0016; and RMSE of 0.16, 0.119, and 0.04, respectively. By directly addressing the forecasting challenges of wind energy, this study supports improved resource management, grid reliability, and operational planning. The findings highlight the effectiveness of hyperparameter-tuned ensemble models, particularly stacking ensembles, in enhancing renewable energy forecasting and advancing global sustainability goals in the future.

## Introduction

Energy forecasting plays a crucial role in managing modern power systems, particularly with the increasing integration of renewable sources, such as hydro, solar, and wind. Although these sources contribute to sustainability, their inherent variability presents challenges to grid stability and reliability^1^. Accurate forecasting is essential for balancing supply and demand, preventing power fluctuations, and optimizing resource utilization in the power industry. Machine learning (ML) and deep learning (DL) have emerged as powerful tools for enhancing forecasting accuracy by analyzing large data sets and identifying complex patterns. These methods enable grid operators to anticipate fluctuations in energy production and demand, facilitating better scheduling, resource allocation, and long-term planning^2^. Additionally, precise forecasting reduces dependence on costly backup power plants, improves energy storage management, and enhances operational efficiency^3^.

In addition to cost efficiency, accurate forecasting contributes to grid stability. By predicting high- and low-demand periods, operators can adjust generation schedules, optimize storage, and implement demand response programs, ensuring a steady power supply even during fluctuations in renewable energy generation^4^. Furthermore, improved forecasting minimizes the reliance on fossil fuels, thereby supporting environmental sustainability by reducing greenhouse gas emissions. This is particularly relevant because regulations continue to mandate increased renewable energy integration^5^. Effective forecasting becomes increasingly critical for grid resilience as renewable energy sources expand. Load projections aid in maintaining stability, optimizing storage, and ensuring that the real-time energy supply aligns with demand^6,7^. Additionally, surplus renewable energy generated during low-demand periods can be stored and utilized during peak demand, enhancing grid efficiency and reducing operational expenses^5,8^. Studies have highlighted the economic benefits of improved forecasting, emphasizing the reduced dependence on expensive backup power plants^9^. Building on prior insights, this study addresses key research gaps by integrating advanced machine learning and deep learning models with real-time, publicly available datasets to improve the accuracy of hourly wind power forecasting. It presents a comprehensive comparative analysis using multiple models and datasets, including a real-time case study from Aralvaimozhi, Tamil Nadu, India. Emphasizing the importance of model selection and systematic hyperparameter tuning, such as grid search with fivefold cross-validation, the study incorporates meteorological features like wind speed and adopts ensemble methods like stacking to develop a robust, scalable forecasting framework. This approach enhances grid reliability, improves energy efficiency, and supports sustainable integration of wind power by addressing its intermittent nature.

## Related works

Traditional load forecasting has long relied on statistical methods, such as Autoregressive Integrated Moving Average (ARIMA) models and linear regression. Although these approaches offer simplicity and ease of visualization, they often struggle to handle nonlinear data and complex patterns arising from renewable energy sources^10,11^. Mystakidis et al. utilized fundamental algorithms like Linear Regression and Decision Trees for energy prediction, leveraging their interpretability and applicability to both regression and classification tasks. However, these models are prone to overfitting if not carefully maintained, making them less reliable in capturing the variability of renewable energy sources^12^.

To overcome these limitations, advanced machine learning (ML) algorithms have been developed for energy forecasting. Random Forest, an ensemble learning method, enhances prediction accuracy by merging multiple decision trees, thereby reducing overfitting^13,14^. Gradient Boosting further refines predictions by iteratively correcting errors from previous models, with XGBoost—a more efficient and scalable variant—offering superior speed and accuracy for complex forecasting tasks^15^. The application of ML in load forecasting has grown significantly because of its ability to process large datasets and capture intricate, nonlinear relationships more effectively than conventional statistical models.

Different ML techniques provide varying advantages depending on the forecasting problems. Support Vector Machines (SVMs) are particularly useful for both linear and nonlinear data, making them applicable to regression and classification tasks^16^. Although Decision Trees and Linear Regression remain straightforward and interpretable, they are susceptible to overfitting, particularly in complex datasets^17^. Random Forest mitigates this by aggregating multiple decision trees, leading to more stable and accurate predictions^18^. Similarly, boosting techniques such as XGBoost have demonstrated efficiency in handling large datasets, allowing models to focus on hard-to-predict samples for improved performance^15^. SVMs, as highlighted by Cortes and Vapnik, provide robustness in both classification and regression tasks, making them well-suited for load forecasting problems that involve diverse data structures^19^.

Deep learning techniques, particularly multilayer perceptron’s (MLPs), have further revolutionized forecasting by capturing intricate nonlinear interactions within datasets. MLPs, which are composed of multiple layers of interconnected neurons, transform input data into increasingly abstract representations, making them highly adaptable across forecasting applications^20^. Their ability to model complex relationships enhances their utility in classification and regression tasks, making them a viable choice for applications ranging from financial modeling to renewable energy forecasting^21^.

Integrating domain-specific knowledge with ML models, such as by incorporating weather data, has further improved forecasting accuracy. Lago et al. and Chen et al. emphasized the importance of external factors, such as wind speed and temperature, in energy predictions. Long Short-Term Memory (LSTM) networks have gained prominence in wind power forecasting owing to their ability to handle sequential time-series data and capture long-term dependencies. Their effectiveness is particularly evident in short-term forecasting, which is crucial for addressing the intermittency of wind energy^22,23^. By learning from historical wind speed data, LSTM models enhance the prediction accuracy even under fluctuating weather conditions^24^. Building on these advancements, this study employed various ML and DL models to forecast wind energy production for a 100 kW (Vestas V90 2000) wind turbine. By leveraging both traditional and advanced forecasting techniques, this study aims to improve the accuracy and reliability of wind energy predictions, contributing to enhanced resource management and grid stability.

Several key research gaps have been identified in the literature and tabulated in Table 1. Conventional statistical models, such as ARIMA and linear regression, frequently fail to adequately capture the intricate, erratic, and nonlinear temporal patterns present in wind energy data. These restrictions make it more difficult to produce accurate projections, particularly in situations where the environment is changing rapidly. Furthermore, many previous studies tend to undervalue the significance of integrating real-time, region-specific data, such as wind data from Aralvaimozhi, Tamil Nadu, which compromises the contextual accuracy and applicability of the forecasts. The absence of thorough comparison studies across a wide variety of machine learning (ML) and deep learning (DL) models that utilize both synthetic and real-time datasets represents another important gap in the field. This makes it more difficult to comprehend the model’s performance with different datasets. Moreover, many studies end with performance reviews that only consider the accuracy of historical data, failing to identify or suggest the best models for upcoming practical forecasting situations. To build reliable, scalable, and context-aware forecasting frameworks that can meet the changing demands of contemporary power systems, these shortcomings must be addressed.

**Table 1:** Summary of literature review on load forecasting techniques.

Author(s)	Model(s) used	Dataset	Findings	Limitations
Taylor et al. ^10^, Hahn et al. ^11^	ARIMA, Linear Regression	Traditional load forecasting data	Simple and easy to visualize, but struggle with non-linear data and complex renewable patterns	Ineffective with non-linear data and complex patterns from renewable sources
Mystakidis et al.^12^	Linear Regression, Decision Trees	Energy prediction datasets	Models are interpretable and applicable to both regression and classification tasks	Prone to overfitting and less reliable for renewable variability
Gellert et al.^13^	Random Forest	Large energy datasets	Enhances prediction accuracy by combining multiple decision trees, reducing overfitting	Computationally intensive, especially when applied to large datasets, compared to simpler base models such as linear regression or decision trees
Chen and Guestrin^15^	XGBoost	Large, complex forecasting datasets	Superior speed and accuracy for complex forecasting tasks, efficient and scalable	Sensitive to hyperparameter settings and can be computationally demanding, especially when compared to simpler base models such as linear regression
Wang et al. ^16^	Support Vector Machines (SVM)	Energy forecasting data	Applicable to both linear and non-linear data for regression and classification tasks	Sensitive to parameter selection can be computationally heavy
Quinlan^17^	Decision Trees	Energy datasets	Straightforward and interpretable	Susceptible to overfitting, especially in complex datasets
Breiman^18^	Random Forest	Energy datasets	More stable and accurate predictions by aggregating multiple decision trees	Requires considerable computational resources
Cortes and Vapnik^19^	SVM	Diverse data structures in load forecasting	Robust in both classification and regression tasks	Computational complexity increases with dataset size
Al Arafat et al.^21^	Multi-Layer Perceptrons (MLPs)	Forecasting datasets	Captures intricate, non-linear interactions; adaptable across forecasting applications	Computationally intensive; requires careful tuning to avoid overfitting
Chen et al.^23^	ML models with weather data integration	Energy datasets with external factors	Incorporating weather data improves forecasting accuracy	Requires accurate external data; forecasting accuracy depends on data quality
Li et al.^24^	LSTM	Historical wind speed data (China)	Effective for short-term wind speed forecasting; captures long-term dependencies and handles time-series data well	Single-region data; lacks cross-regional validation

Based on the research gap identified, the main objective of this study is to present significant advancements in renewable energy forecasting, with an emphasis on wind power generation. This study developed a comprehensive hybrid forecasting framework that balances local applicability with global relevance by integrating region-specific data from the Aralvaimozhi corridor (sourced from Renewables Ninja) and publicly available datasets from Kaggle. A diverse set of machine learning and deep learning models was rigorously assessed, and their performance was benchmarked under various scenarios. These findings underscore the superior predictive capability of ensemble models, particularly Random Forest and XGBoost, when trained on high-resolution, real-time datasets. Furthermore, the inclusion of a stacking ensemble approach enhanced the validation process and strengthened the accuracy of future forecasts. The exceptional forecasting precision achieved demonstrates the effectiveness of the models in supporting energy management and real-time grid integration. This study contributes to improved grid stability, optimized operational planning, and more efficient supply–demand balancing and dispatch strategies. By focusing on the wind-abundant Aralvaimozhi corridor, this study delivers region-specific insights of considerable value to policymakers, energy planners, and wind farm operators in Tamil Nadu.

## Methodology

Machine Learning (ML) and Deep Learning (DL) models have emerged as powerful tools for enhancing the accuracy and efficiency of wind energy forecasting. Traditional numerical weather prediction models and statistical approaches often struggle to account for the dynamic atmospheric conditions and nonlinear dependencies that are inherent in wind data. In contrast, ML and DL techniques offer data-driven solutions that improve predictive capabilities by uncovering complex patterns in historical wind speed and energy-generation data. This study employs a combination of machine learning models—XGBoost, Random Forest, Decision Trees, Linear Regression, and K-Nearest Neighbors (KNN)—along with deep learning models such as Multi-Layer Perceptron (MLP) and Long Short-Term Memory (LSTM) networks to forecast wind energy production. The obtained results were compared with those in the existing literature to assess the performance improvements.

## Research region and meteorological information

This study focuses on the Aralvaimozhi Pass, a natural wind corridor in the Kanyakumari District of Tamil Nadu, India. This region, situated at the southernmost end of the Western Ghats, is characterized by consistently high wind speeds of up to 25 m/s, making it an ideal location for wind energy generation.

**Fig. 1:**
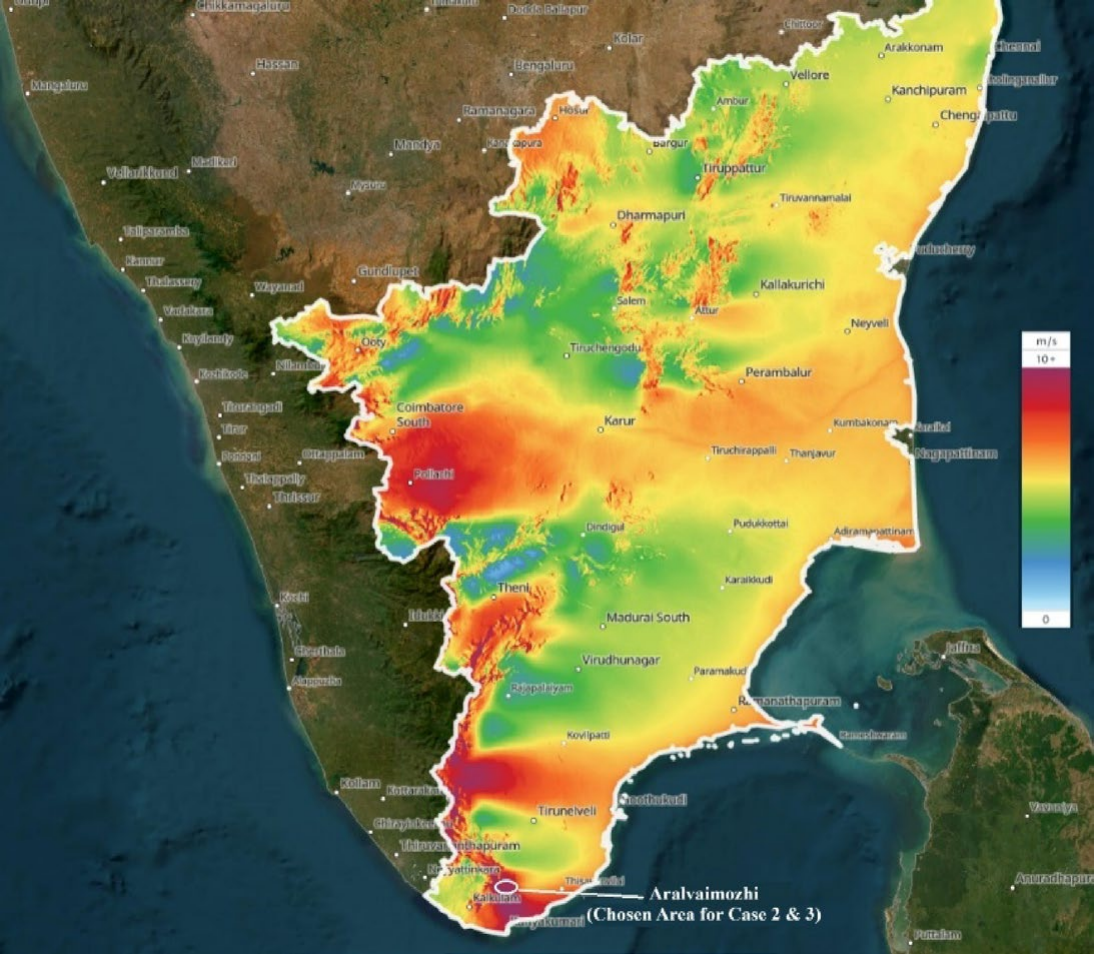
Wind speed map of Aralvaimozhi, southern Tamil Nadu region.

The broad, elevated topography facilitates strong wind currents, ensuring reliable energy production annually. Additionally, the region benefits from a low population density, proximity to existing power transmission infrastructure, and favorable government policies that support renewable energy investments. The dataset used in this study spanned one year and was sourced from the Modern-Era Retrospective Analysis for Research and Applications (MERRA-2). Figure 1 illustrates the Wind speed map of Tamil Nadu, highlighting the Aralvaimozhi region. Base map obtained from the Global Wind Atlas version 3.3, a free, web-based application developed, owned, and operated by the Technical University of Denmark (DTU), in partnership with the World Bank Group, using data provided by Vortex and funding from ESMAP. Available at https://globalwindatlas.info.

The colour gradient depicts varying wind activity levels, with green, yellow, and related tones indicating moderate to strong winds^25^. Aralvaimozhi, identified as a high wind speed zone in the figure, was specifically selected for wind power forecasting in Cases 2 and 3 of this study.

### Work flow of the proposed methodology

**Fig. 2:**
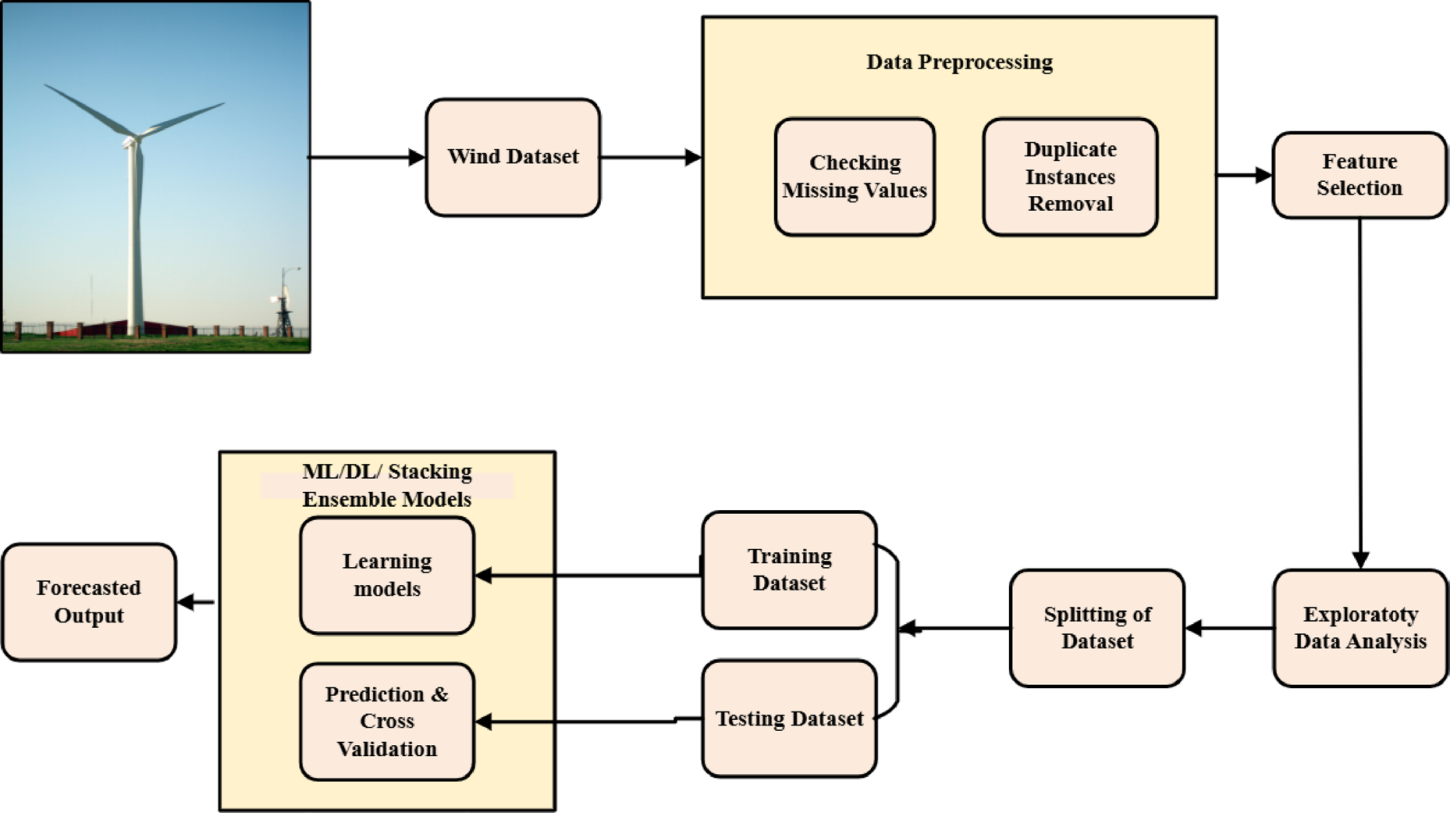
Workflow model of the proposed methodologies.

The methodology follows a structured workflow, beginning with data preprocessing, which involves selecting key features, handling missing values, and eliminating duplicate records. Exploratory Data Analysis (EDA) was then conducted to visualize the data distributions, identify trends, and detect outliers. To ensure model robustness, 70% of the dataset was allocated for training, whereas the remaining 30% was used for testing and evaluation. During the machine learning regression phase, predictive models are trained on historical data and validated using cross-validation techniques. The trained models then used the input wind data to estimate future energy production values. This approach ensures accurate wind energy forecasts, which are critical for optimizing turbine operation and grid stability. The models were implemented using Python on the Jupyter Notebook platform. Figure 2 provides an overview of the workflow, detailing the data flow from the preprocessing to the prediction.

### Predictive models

This study evaluated various ML and DL models to determine the most effective approach for wind energy forecasting.

- *Linear Regression*, the simplest model, assumes a linear relationship between wind speed and turbine output. Although it offers interpretability, its inability to handle nonlinear dependencies limits its effectiveness for complex wind patterns^26–28^.
- *Decision Trees* provide more flexibility by capturing decision rules based on wind speed variations. However, they are prone to overfitting, particularly in small datasets^17^.
- *Random Forest and Extra Trees*, ensemble-based algorithms, enhance predictive accuracy by averaging multiple decision trees, reducing overfitting, and improving generalization. Extra Trees further increases randomness, which strengthens the model robustness^18,28–30^.
- *Gradient Boosting and XGBoost* employ boosting techniques to sequentially refine predictions. Gradient Boosting minimizes loss functions at each step, ensuring improved accuracy, whereas XGBoost prioritizes computational efficiency and regularization, mitigating overfitting^15,31^.
- *K-Nearest Neighbors (KNN)* offers a non-parametric alternative by basing predictions on the average of neighboring data points. Although effective in identifying local trends, KNN is computationally expensive for large datasets and sensitive to noise^32–34^.
- *AdaBoost*, another boosting technique, enhances weak learners by assigning greater importance to misclassified instances and iteratively refining the model performance. However, careful tuning is required to prevent overfitting^35,36^.

Deep learning models further improve forecasting accuracy by capturing complex temporal dependencies.

- *Multi-layer perceptrons (MLP)* utilize multiple hidden layers to transform raw input features into high-level abstractions. Through backpropagation, MLPs adjust the weights and biases to optimize predictions, making them effective for nonlinear problems. However, their black-box nature and computational demands require careful interpretation and optimization^37,38^.
- *Long Short-Term Memory (LSTM)* networks are particularly suited for wind forecasting because they are designed to capture long-term dependencies in time-series data. Unlike traditional neural networks, LSTMs retain important past information, allowing them to model the sequential patterns of wind speed and turbine output. This capability enhances the prediction accuracy, particularly under volatile weather conditions^39–41^.
- *Stacking Ensemble Model*: To improve the prediction accuracy and resilience of wind power projections, the forecasting framework created in this study incorporates several sophisticated ensemble learning algorithms. Random Forest was used because of its overall stability and ability to efficiently handle noisy and varied inputs. To improve the model’s prediction ability in situations with complicated patterns, XGBoost was used to capture complex nonlinear interactions within the dataset. Faster model training and inference without compromising accuracy are made possible by LightGBM’s excellent computational efficiency and capacity to handle large-scale datasets. Additionally, by adding extra randomization during feature splits, the Extra Trees technique was presented to improve model diversity and reduce overfitting. A Stacking Ensemble approach was used to combine the effectiveness of several separate models, combining their outputs via a meta-learner to generate the final forecast. When used on real-time datasets for wind power prediction and future energy generation scenarios, this ensemble technique not only improves the overall forecasting accuracy but also performs better^29,41^.
- *Ensemble Model Construction*: To enhance prediction robustness and accuracy, a Stacking Ensemble framework was employed. This method integrates the predictive capabilities of multiple diverse models like Random Forest, XGBoost, Extra Trees, and LightGBM as base learners, and combines their outputs using a meta-learner, typically a Linear Regression model in this study.

In the stacking ensemble approach, the training dataset is partitioned into *K* folds (with *K* = 5 used in this study). For each base learner, K-fold cross-validation is performed, and out-of-fold (OOF) predictions are generated for each training fold. These OOF predictions are then concatenated to form a new feature matrix Z, as mentioned in Eq. (1) which serves as the input for training the meta-learner. After generating OOF predictions for all base learners, the final meta-feature matrix $Z \in {\mathbb{R}}^{n\times k}$ is constructed, where n is the number of training samples and k is the number of base models. The meta-learner is subsequently trained on this matrix Z using the corresponding ground-truth labels y, allowing it to learn how to optimally combine the outputs of the base learners for final prediction as mentioned in Eq. (2).

The mathematical formulation for Stacking Ensemble model is

Let,

$X \in {\mathbb{R}}^{n \times d}$- input features (n samples, d features)

$y \in {\mathbb{R}}^{n}$- target output vector

${f}_{i}$- ith base learner model (RF, XGBoost, Extra tree and LightGBM)

$y_{i}^{ \wedge } = f_{i} \left( X \right) \in {\mathbb{R}}^{n}$- prediction from the ith base learner

k- number of base learners

$Z\in {\mathbb{R}}^{n\times k}$- Meta-feature matrix formed by stacking predictions from all base learners

$g(\cdot)$- Meta-learner (Linear regression)

Then the meta-feature matrix Z is given by

1 $$Z = \left[ {f_{1} \left( X \right), f_{2} \left( X \right), \ldots f_{k} \left( X \right)} \right] \in {\mathbb{R}}^{n \times k}$$

where each $y_{i}^{ \wedge } \in {\mathbb{R}}^{n}$ is the final prediction from the meta-learner g,

2 $$y_{final}^{ \wedge } = g\left( Z \right) = g\left( {f_{1} \left( X \right), f_{2} \left( X \right), \ldots f_{k} \left( X \right)} \right)$$

- *Pseudocode for Stacking Ensemble Training*

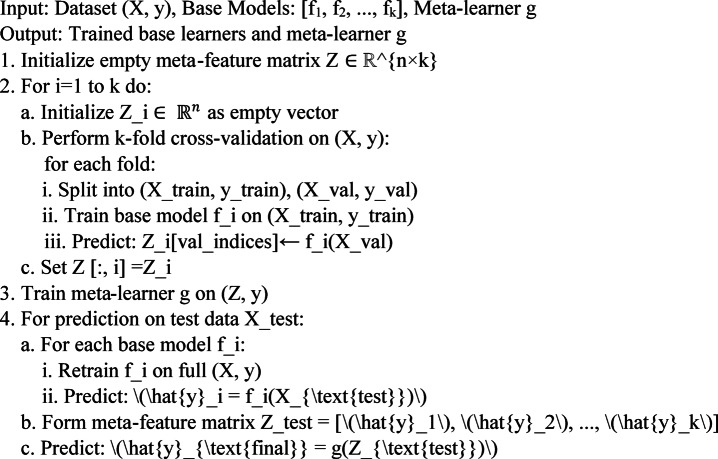

**Fig. 3:**
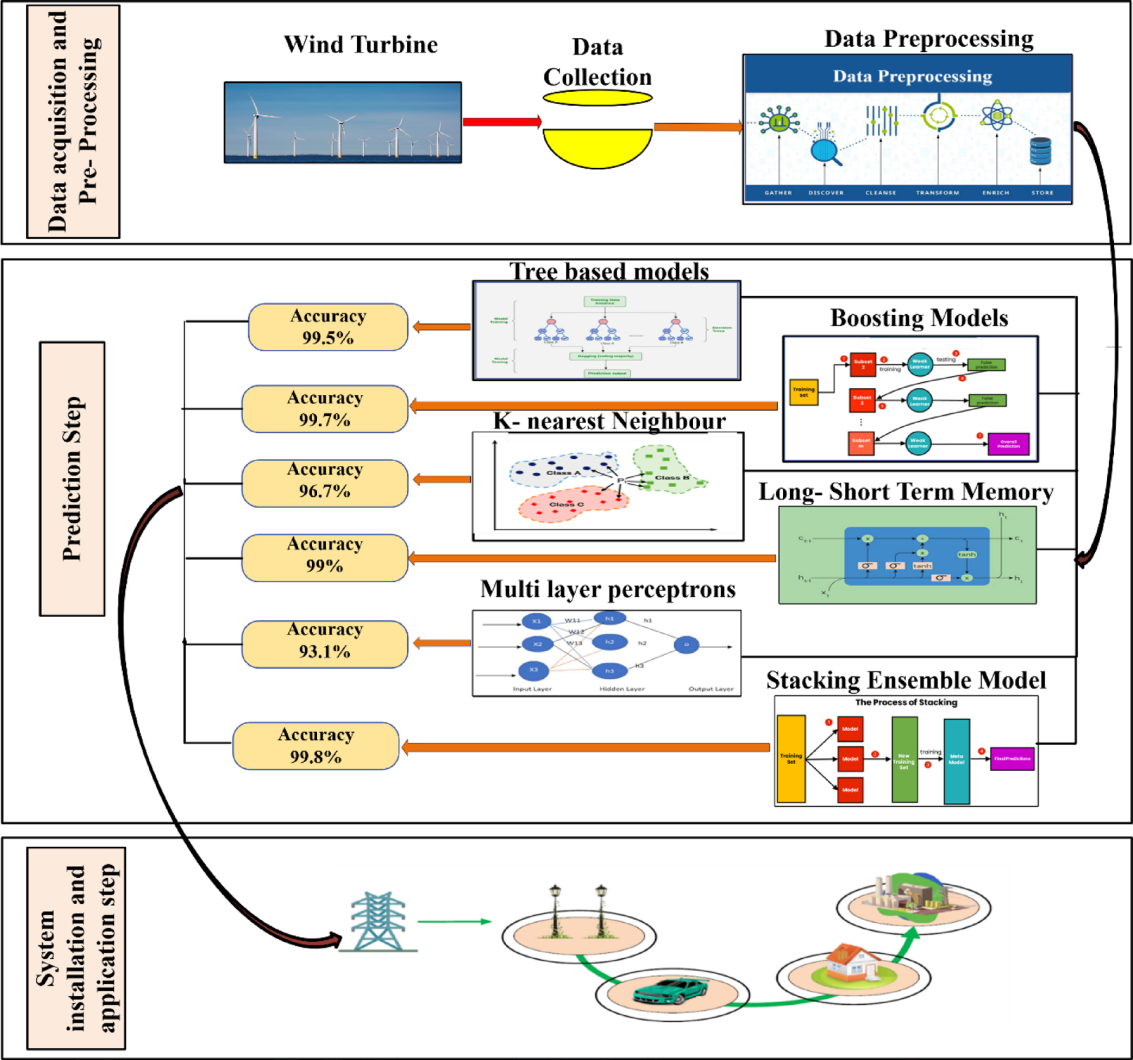
Machine learning framework for wind energy prediction and application.

Figure 3 outlines the complete data processing pipeline, from the initial data collection and preprocessing to the model training and evaluation. The integration of ML, DL, and stacking ensemble techniques ensures a comprehensive approach to wind energy forecasting, providing reliable insights for optimizing turbine efficiency and energy grid management.

### Computational complexity of ML models

In the academic literature and practical applications, the computational complexity of machine learning (ML) models is often presented as a theoretical approximation. However, real-world execution times are influenced by multiple factors, including hardware specifications, feature dimensionality, dataset size, and algorithmic optimization. While theoretical complexity provides insights into how algorithms scale, it does not fully capture practical considerations, such as memory usage, constant factors, and the benefits of hardware acceleration, including parallelization^38,42^. Additionally, the efficiency of ML models is affected by the quality and quantity of data, as well as hardware-specific optimizations, such as GPU acceleration and distributed computing frameworks^43,44^. The practical computational complexity of a machine learning model is determined by both the characteristics of the algorithm and the underlying hardware configuration. Factors such as the number of CPU cores, availability of GPUs, and parallel execution strategies significantly affect the execution times. Moreover, model-specific aspects, including regularization techniques, convergence criteria, and hyperparameter tuning, contribute to variations in the actual performance^45^. Importantly, although ensemble tree-based methods like Random Forest and XGBoost require iterative model training and can seem computationally demanding during hyperparameter tuning, their prediction (inference) time is fast, and they are far more computationally efficient than deep learning models such as LSTM and MLP in typical forecasting scenarios. Moreover, their structure allows for efficient parallel processing, making them highly suitable for practical applications where periodic retraining is acceptable and real-time inference is required. Their inclusion in the stacking ensemble was therefore motivated by their predictive strength and robustness, not at the cost of impractical computational overhead.

The execution time of the ML and DL models depends on several factors, including the dataset size, implementation efficiency, and hardware capabilities. Although theoretical complexity provides a useful comparison framework, real-world performance varies owing to optimizations such as mini-batch processing, vectorized computations, and parallel execution on GPUs or distributed computing platforms. Selecting the appropriate model requires balancing computational efficiency with predictive accuracy, ensuring that the chosen approach aligns with the problem constraints and the available computational resources^46^.

### Model training and optimization

Every machine learning and deep learning algorithm utilized in this study underwent thorough hyperparameter adjustment to improve the predictive performance and guarantee a fair comparison across all models, as detailed in Table 2. The number of estimators, maximum tree depth, learning rate, and subsampling ratio were among the crucial hyperparameters that were adjusted for ensemble models such as Random Forest, XGBoost, AdaBoost, and Gradient Boosting by combining grid search and random search techniques. Specifically, a grid search with fivefold cross-validation was employed to exhaustively explore hyperparameter combinations and assess model generalizability across different data splits. The tuning process for deep learning models, such as Multi-Layer Perceptron (MLP) and Long Short-Term Memory (LSTM), focuses on determining the ideal set of hidden layers, neurons per layer, activation functions, dropout rates, batch size, and learning rate.

**Table 2:** Optimized hyperparameters for machine learning and deep learning models.

Model	Hyperparameter search space	Optimized hyperparameters
Random forest	n_estimators: [100, 200, 300]; max_depth: [10, 20, 30]; min_samples_split: [2, 5]	n_estimators = 200; max_depth = 30; min_samples_split = 2
Gradient boosting	n_estimators: [100, 200]; learning_rate: [0.01, 0.1]; max_depth: [3, 5]	n_estimators = 200; learning_rate = 0.1; max_depth = 5
Decision tree	max_depth: [5,10,20]; criterion: [‘squared_error’, ‘friedman_mse’]	max_depth = 20; criterion = ‘squared_error’
K-nearest neighbors	n_neighbors: [3, 5, 7]; weights: [‘uniform’, ‘distance’]	n_neighbors = 7; weights = ‘distance’
AdaBoost	n_estimators: [50, 100, 200]; learning_rate: [0.01, 0.1, 1]	n_estimators = 200; learning_rate = 0.1; loss = ‘exponential’
XGBoost	n_estimators: [100, 200, 300]; learning_rate: [0.01, 0.05, 0.1]; max_depth: [3, 6, 9]	n_estimators = 300; learning_rate = 0.1; max_depth = 6
Extra trees classifier	n_estimators: [100, 200, 300, 500], max_depth: [10, 20, 30, None], min_samples_split: [2, 5, 10],	n_estimators = 300; max_depth = 20; min_samples_split = 2
MLP	units: [64, 128]; dropout: [0.2, 0.3]; learning_rate: [0.001, 0.01]; batch_size: [32, 64]; epochs: [50, 100]	units = 128; num_layers = 2; dropout = 0.3; optimizer = ‘adam’; learning_rate = 0.01; batch_size = 64; epochs = 100
LSTM	units: [64, 128]; dropout: [0.2, 0.3]; learning_rate: [0.001, 0.01]; sequence_length: [30, 50]; batch_size: [32, 64]; epochs: [50, 100]	units = 128; num_layers = 2; dropout = 0.3; optimizer = ‘adam’; learning_rate = 0.001; batch_size = 64; epochs = 100; sequence_length = 50

To avoid overfitting and guarantee the robustness of the chosen hyperparameters, cross-validation techniques, including fivefold cross-validation integrated within the grid search, were used throughout the tuning process. Based on the validation performance, the final configurations were selected, significantly enhancing the model accuracy and reliability across both the real-time dataset and future forecasting scenarios. Grid search with cross-validation is a widely adopted technique, it was employed in this study to ensure consistency and fairness across all models being compared. Each model’s hyperparameters were tuned using an exhaustive grid search strategy with fivefold cross-validation, allowing us to maximize model generalizability and minimize overfitting. This comprehensive hyperparameter tuning strategy played a critical role in achieving the high R^2^ scores and low error metrics reported in the results, ultimately boosting the overall performance of the forecasting framework.

### Evolution metrices used for forecasting electric power from wind source

The performance of each model was rigorously assessed using a comprehensive set of evaluation metrics. The Mean Squared Error (MSE) was employed as to evaluate the average squared differences between the predicted and actual values, providing insight into the model’s accuracy while penalizing larger errors more heavily.

3 $$MSE = \frac{1}{n}\mathop \sum \limits_{i = 1}^{n} \left( {y_{a} - y_{\Pr } } \right)^{2}$$

The average magnitude of errors was evaluated using the Mean Absolute Error (MAE), which provides a straightforward measure of forecast accuracy.

4 $$MAE = \frac{1}{n}\mathop \sum \limits_{i = 1}^{n} \left| {y_{a} - y_{\Pr } } \right|$$

The Root Mean Square Error (RMSE), another widely used metric for regression models, was calculated as the square root of the mean squared differences between the actual and predicted values. RMSE is particularly valuable when minimizing large errors is critical, as it penalizes larger discrepancies more heavily because of the squaring process. A lower RMSE indicates a better fit between the model and data.

5 $$RMSE = \sqrt {\frac{1}{n}\mathop \sum \limits_{i = 1}^{n} \left( {y_{a} - y_{\Pr } } \right)^{2} }$$

Additionally, the model’s capacity to capture the variance in the output was assessed using the R^2^ score, where values closer to 1 signified a stronger fit. This multifaceted evaluation approach ensures a thorough understanding of the model’s strengths and weaknesses, enabling informed decisions regarding model selection and enhancement.

6 $$R^{2} = 1 - \frac{{\mathop \sum \nolimits_{i = 0}^{n} \left( {y_{a} - y_{pr} } \right)^{2} }}{{\mathop \sum \nolimits_{i = 0}^{n} \left( {y_{a} - y^{ - } } \right)}}$$

where *n*: the total number of data points (observations). ${y}_{a}$: The actual observed values of the dependent variable at index i. ${y}_{pr}$: The predicted values of the dependent variable from the model at index i. ${y}^{-}$: The mean of the observed values.

### Evaluating wind power potential in southern India

Forecasting future electricity generation is essential for maintaining grid stability, optimizing costs, and maximizing the integration of renewable energy sources into the grid. Renewable energy resources currently contribute significantly to global power requirements, with wind energy playing a crucial role. However, owing to its intermittent nature, predicting wind energy generation requires accurate forecasts based on weather conditions^47^. Precise forecasting helps to balance supply and demand, preventing power surpluses or shortages that could destabilize the system^48^.

Wind patterns vary across regions, making site selection a critical factor in ensuring efficient wind energy generation and aligning production with the anticipated energy demand. In this study, the Aralvaimozhi region in southern India was chosen as the primary location for analysis. Since 2019, noticeable seasonal variations in wind speed have been observed in Aralvaimozhi, Tamil Nadu, India. Typically, wind speeds peak during the southwest monsoon season (June to September) and then decline in winter. These variations, including periodic oscillations, have a significant influence on local wind power generation.

Key factors, such as wind speed range (measured in m/s) and temporal wind speed fluctuations, were analyzed. Studies by the Council on Energy, Environment and Water, New Delhi, indicate a modest but notable trend of decreasing wind speeds in the area, which could impact the future potential of wind energy production (Council on Energy, Environment and Water n.d.). Although the exact rate of decline varies, some data suggest that the average wind speeds in parts of southern Tamil Nadu may be decreasing by as much as 0.6% annually, potentially reducing wind farm performance (https://www.ceew.in/). For load forecasting in this study, wind speed fluctuations between 3 and 15 m/s were considered, as referenced in previous studies^46,47^.

**Fig. 4:**
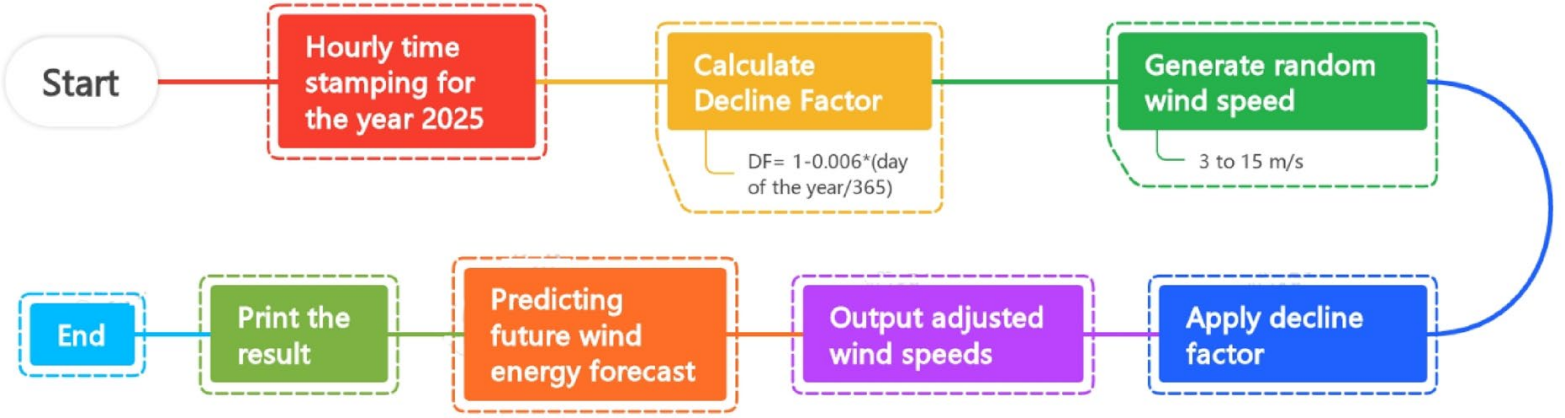
Flow chart for assessing the future wind power generation.

The procedure for future forecasting wind energy for the entire year of 2025 is shown in Fig. 4. Hourly time stamping for the full year was the first step, and then a decline factor (DF = 1 − 0.006 × day/365) was calculated to modify the wind speed according to seasonal patterns. The decline factor was used to adjust the randomly produced wind speeds, which ranged from 3 to 15 m/s. The future wind energy output was predicted using the modified wind speeds, and the results were printed before the procedure was completed. The flowchart illustrates a methodical approach to model and examine annual wind energy trends.

## Results and discussion

This section evaluates the performance of various machine learning (ML) and deep learning (DL) models for wind energy prediction in three different scenarios.

- *Case 1*: The Kaggle wind turbine SCADA dataset was used for energy forecasting.
- *Case 2*: Real-time wind data from Aralvaimozhi, located in southern Tamil Nadu, India, sourced from Renewables Ninja, were employed for predictive analysis.
- *Case 3*: Focuses on forecasting future wind energy generation using the top-performing models identified in previous cases.

### Case 1: wind turbine SCADA dataset evaluation

In this study, the proposed ML and DL models were evaluated using the Wind Turbine SCADA Dataset from Kaggle, which contains 50,530 samples, including 2,030 missing data instances. The dataset was split into 70% for training and 30% for testing to ensure a robust model evaluation.

To benchmark the performance, the results obtained from the models were compared with the findings of Karaman^49^, who conducted a similar study using the same wind turbine SCADA dataset (https://www.kaggle.com/datasets/berkerisen/wind-turbine-scada-dataset). A comparative analysis was conducted, and the results are presented in Tables 3 and 4.

**Table 3:** Performance metrics evolution for Kaggle wind turbine SCADA dataset by Karaman^49^.

ML models	Performance evaluation on training data set				Performance evaluation on testing data set			
	MAE	MSE	RMSE	R^2^	MAE	MSE	RMSE	R^2^
ANN	0.0224	0.0057	0.0757	0.9345	0.0245	0.006	0.0787	0.9301
CNN	0.0218	0.0054	0.0732	0.9388	0.0235	0.0055	0.0742	0.9378
RNN	0.0196	0.0038	0.0615	0.9567	0.0218	0.0043	0.0656	0.9514
LSTM	0.0179	0.0027	0.0517	0.9694	0.0209	0.0038	0.0614	0.9574

**Table 4:** Performance metrices evolution by the proposed machine language algorithms for the Kaggle wind turbine SCADA dataset.

ML models	Performance evaluation on training data set				Performance evaluation on testing data set			
	MAE	MSE	RMSE	R^2^	MAE	MSE	RMSE	R^2^
Linear Regression	0.05	0.05	0.05	0.835	0.05	0.05	0.05	0.846
MLP	0.035	0.034	0.041	0.89	0.031	0.032	0.036	0.889
AdaBoost	0.032	0.032	0.04	0.90	0.028	0.030	0.035	0.893
Gradient Boosting	0.017	0.015	0.03	0.950	0.009	0.011	0.017	0.948
Decision Tree	0.003	0.009	0.014	0.961	0.003	0.010	0.015	0.953
Extra Trees	0.0051	0.002	0.009	0.99	0.004	0.007	0.011	0.962
Random Forest	0.004	0.0012	0.008	0.996	0.002	0.003	0.006	0.972
K-Nearest Neighbours	0.0087	0.004	0.02	0.99	0.010	0.001	0.002	0.977
XGBoost	0.0083	0.003	0.013	0.99	0.009	0.004	0.023	0.980

**Fig. 5:**
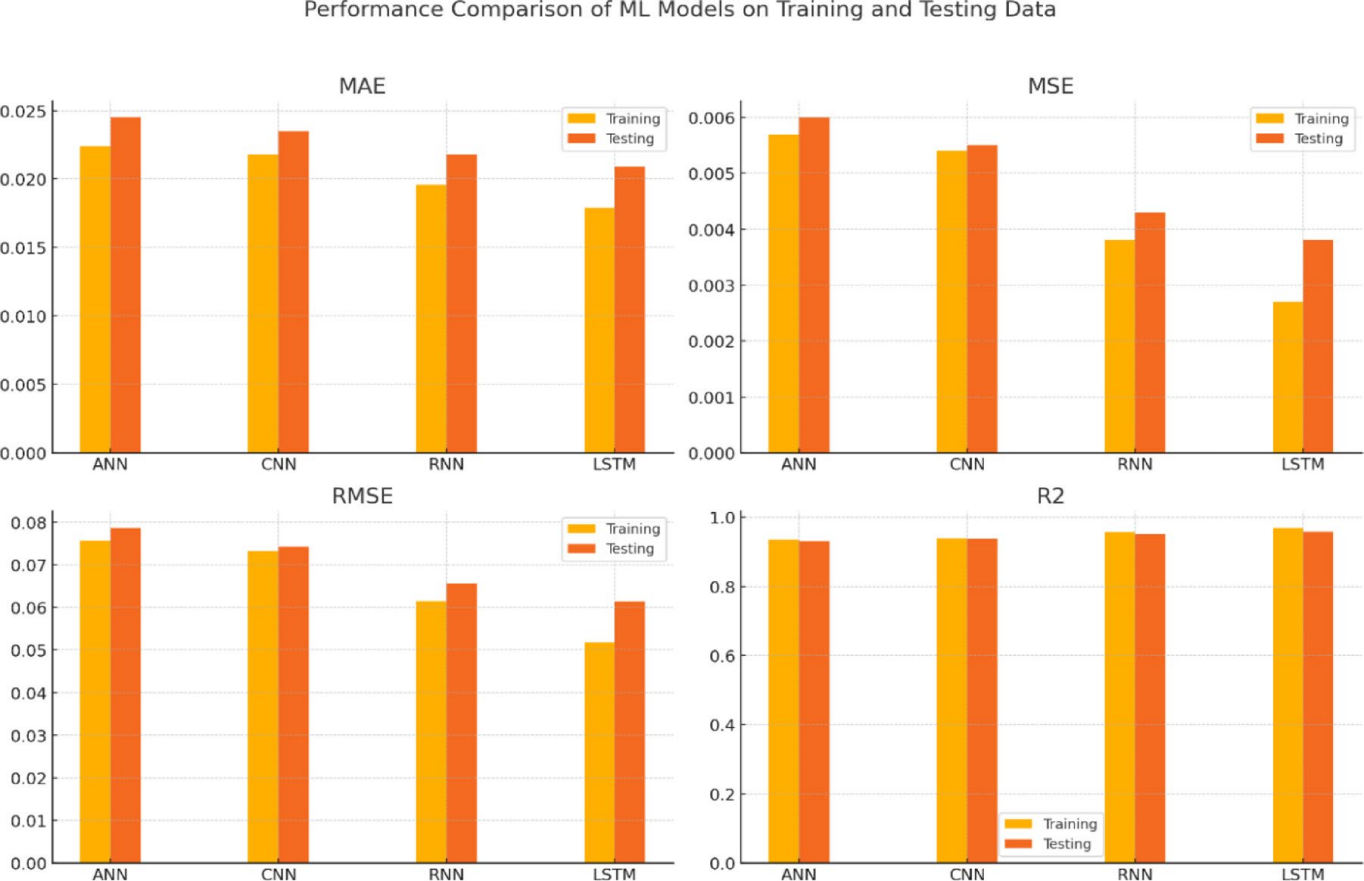
Performance comparison of ML/DL models on training and testing data.

Table 3 and Fig. 5 summarize the model performance reported by Karaman (2023) for various deep learning approaches, including Artificial Neural Networks (ANN), Convolutional Neural Networks (CNN), Recurrent Neural Networks (RNN), and Long Short-Term Memory (LSTM). Karaman (2023) identified LSTM as the top-performing model, achieving the lowest Mean Absolute Error (MAE), Mean Squared Error (MSE), and Root Mean Squared Error (RMSE), along with a high R^2^ value of 0.9694. While slightly trailing LSTM, RNN and CNN also demonstrated strong predictive capabilities, effectively capturing the temporal and nonlinear patterns in the data.

**Fig. 6:**
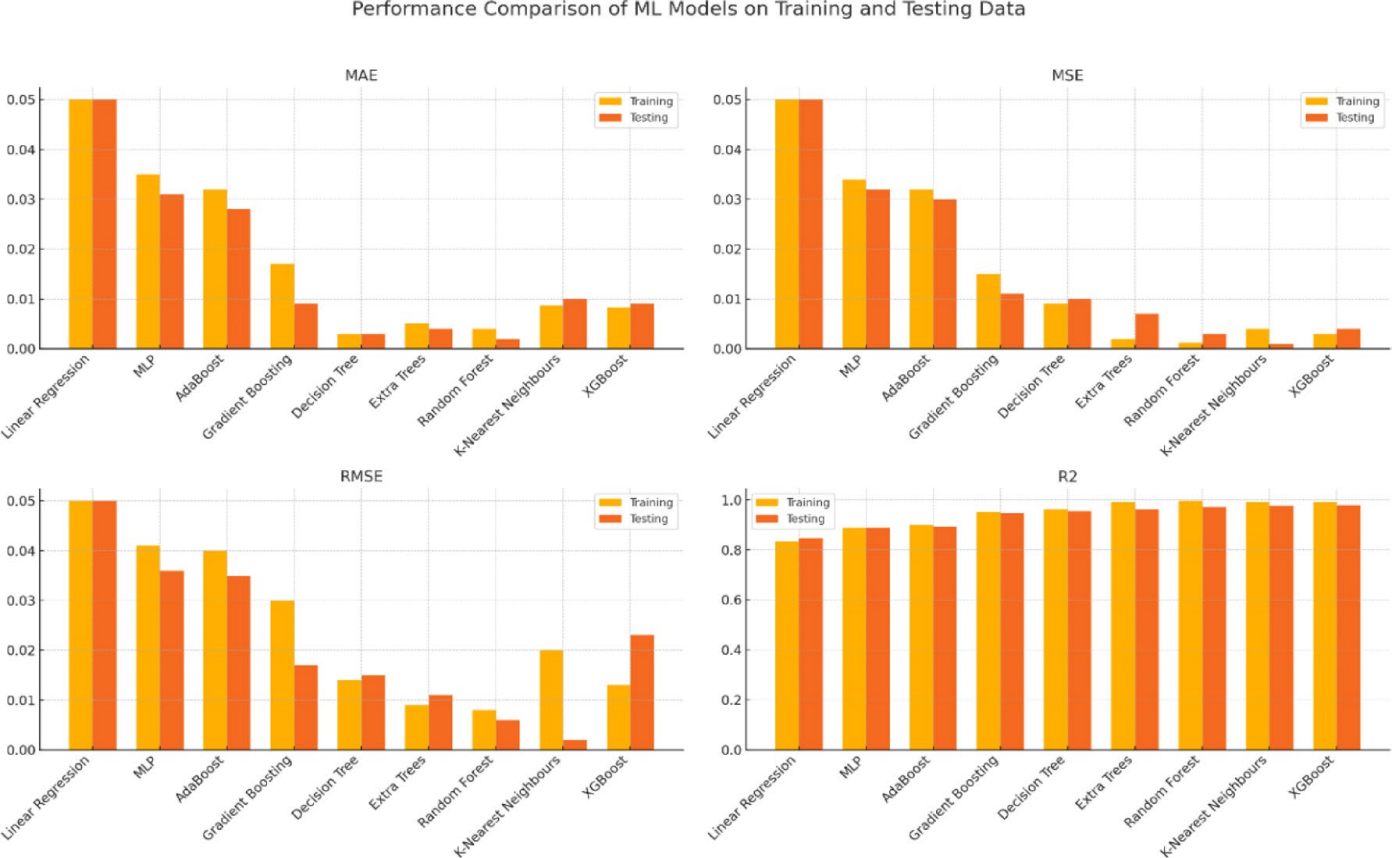
Performance comparison of proposed tuned ML/DL models on training and testing data.

Further performance improvements are shown in Table 4 and Fig. 6, which evaluate the accuracy of the proposed machine learning models. On the testing dataset, ensemble techniques such as Random Forest, Extra Trees, and XGBoost achieved notably high R^2^ values of 0.972, 0.962, and 0.980, respectively, highlighting their ability to handle complex nonlinear interactions while maintaining minimal error metrics. While Adaboost and Linear Regression yielded comparatively lower R^2^ values (0.846 and 0.893, respectively), models such as K-Nearest Neighbors and Decision Trees also demonstrated competitive results.

Overall, the comparison revealed that while LSTM excels in time-series forecasting (Table 3), ensemble methods—particularly Random Forest, Extra Trees, and XGBoost—exhibit superior accuracy and robustness (Table 4). These findings underscore the importance of selecting advanced models to enhance the accuracy of renewable energy forecasting and ensure more reliable wind energy predictions for grid stability and efficiency.

### Case 2: real-time wind data analysis

**Fig. 7:**
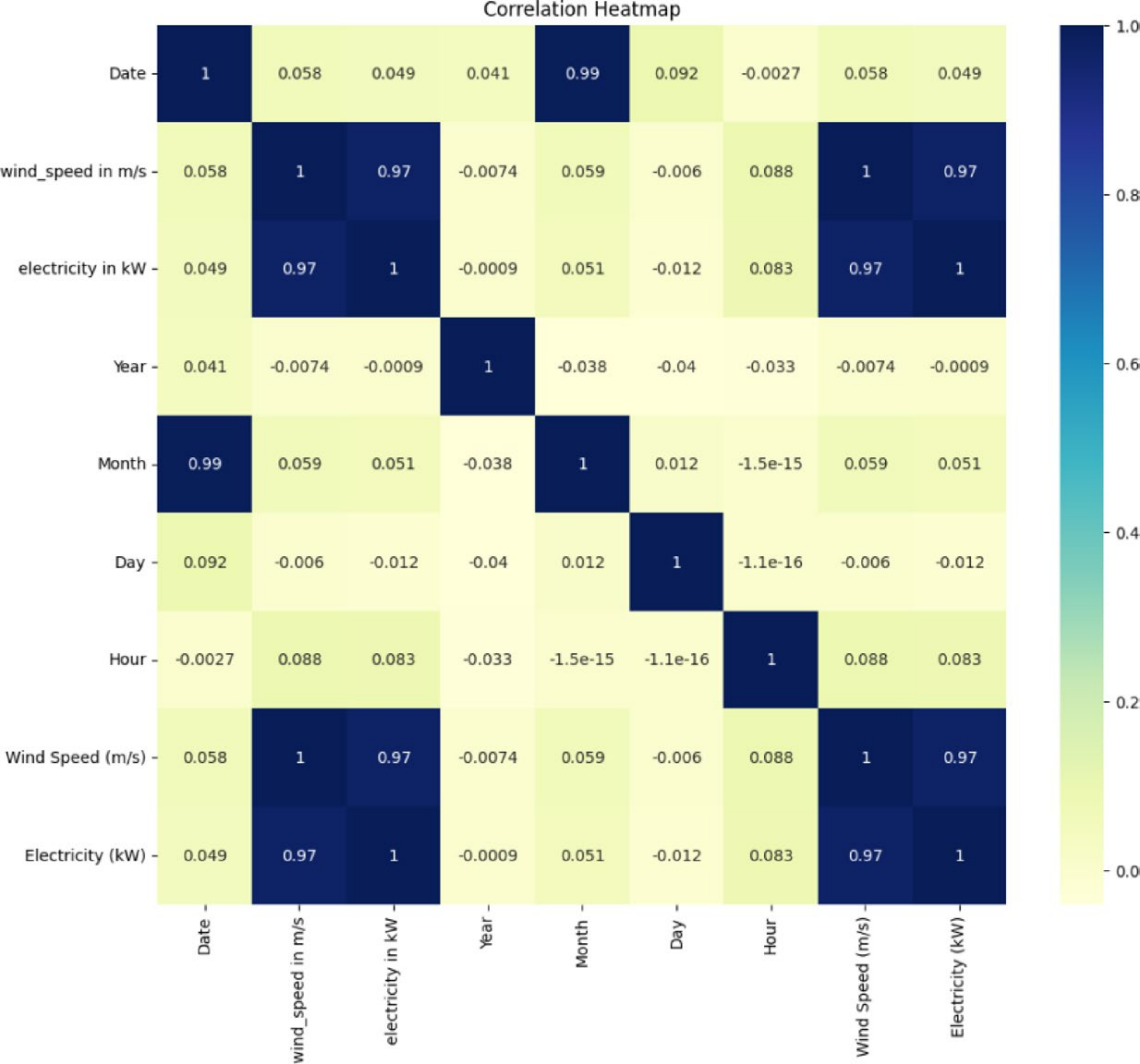
Correlation heat map for the Aralvaimozhi data set.

Case 2 used a real-time dataset from Aralvaimozhi, located in southern Tamil Nadu, India, sourced from Renewables Ninja, for wind energy prediction. The dataset comprised 8,650 samples, including attributes such as date, time, wind speed, and electricity generation. A correlation heatmap (Fig. 7) illustrates the relationships between these variables, revealing a strong positive correlation (0.97) between the wind speed and power generation. This indicates that higher wind speeds lead to increased electricity output, making wind speed the primary factor influencing power output. Additionally, the date and month exhibited a high correlation (0.99) because the month was derived from the date. However, time-based variables such as the year, day, and hour have a minimal impact on power generation and wind speed.

#### Statistical analysis

**Fig. 8:**
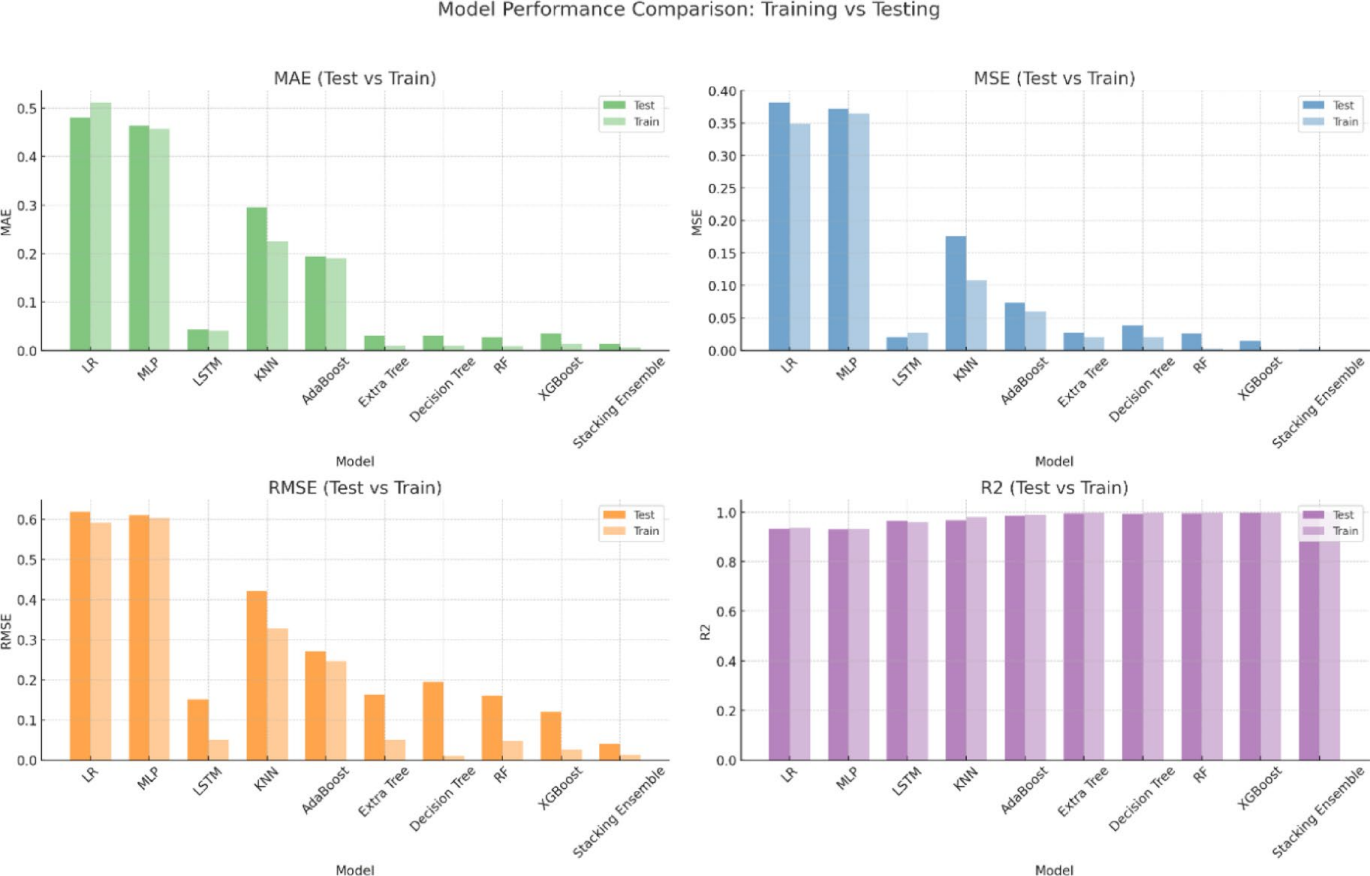
Various ML/DL/Ensemble model performance over training and testing data.

The performance comparison of various models shown in Table 5 and Fig. 8 reveals some clear patterns in terms of accuracy, generalization, and robustness. Linear Regression (LR), which starts with the baseline models, performs the worst on all criteria. The test R^2^ of 0.935 and MAE of 0.481 indicate that it has difficulty capturing the intricacy of the data. Similarly, the Multi-Layer Perceptron (MLP) marginally outperforms LR, suggesting that this dataset may include complicated nonlinear interactions that make a basic neural network architecture insufficient.

**Table 5:** Comparison of statistical analysis of various machine learning models.

MODEL	MAE		MSE		RMSE		R^2^	
	Test	Train	Test	Train	Test	Train	Test	Train
LR	0.481	0.511	0.381	0.349	0.617	0.591	0.935	0.937
MLP	0.463	0.457	0.372	0.364	0.61	0.603	0.931	0.933
LSTM	0.043	0.04	0.02	0.027	0.151	0.05	0.9635	0.9601
KNN	0.295	0.225	0.176	0.107	0.42	0.327	0.967	0.98
AdaBoost	0.194	0.19	0.073	0.06	0.271	0.246	0.986	0.989
Extra tree	0.03	0.01	0.027	0.02	0.163	0.049	0.995	0.997
Decision tree	0.03	0.01	0.038	0.02	0.194	0.01	0.993	0.998
RF	0.027	0.009	0.026	0.002	0.16	0.047	0.995	0.999
XGBoost	0.035	0.014	0.014	0.001	0.119	0.026	0.997	0.999
Stacking ensemble	0.014	0.006	0.0016	0.0002	0.04	0.012	0.998	0.9997

The LSTM model, however, showed a significant improvement, with a low-test MAE of 0.043 and a strong R^2^ of 0.9635. LSTMs are well-suited for time-series or sequence-based data, which suggests that the dataset might have temporal dependencies (for example, solar irradiance or weather variations over time). The model generalizes well, as the training and test scores are close, indicating no major overfitting. The K-Nearest Neighbors (KNN) model performed moderately, with a test MAE of 0.295 and an R^2^ of 0.967. Although it performs better than the linear models, it is still outperformed by the tree-based ensembles. KNN’s sensitivity of KNN to data scaling and feature relevance may limit its effectiveness here. AdaBoost offers a noticeable performance improvement when switching to ensemble approaches, with a test MAE of 0.194 and R^2^ of 0.986. AdaBoost outperformed the solo models in handling complicated patterns and successfully reduced bias. Good generalization is demonstrated by the closeness of the training and testing errors.

Tree-based models, such as Decision Trees and Extra Trees, further improve the performance. Extra Trees’ fantastic R^2^ of 0.995 and great test MAE of 0.03 show how effectively this approach captures the data distribution. Although they perform well, the slightly higher RMSE of Decision Trees than that of extra trees suggests that their forecasts may vary slightly more. The performance was further improved using Random Forest (RF), which yielded an R^2^ of 0.995 and a low-test MAE of 0.027. It is a dependable option because it may minimize overfitting by averaging many decision trees. With a test MAE of 0.035 and an outstanding R^2^ of 0.997, XGBoost, which is well-known for its effectiveness and predictive capacity, marginally outperformed RF and ranked among the best in this assessment.

Finally, the most effective strategy in this investigation is the Stacking Ensemble model, which significantly improves the forecast accuracy and resilience of wind power estimates. To guarantee a thorough and dependable performance, the forecasting framework integrates several sophisticated ensemble learning techniques. Random Forest is ideal for complicated environmental datasets because of its exceptional stability and capacity to handle various noisy data inputs. XGBoost is used to capture complicated nonlinear correlations in the data, improving the model’s prediction power, particularly in situations where patterns are extremely detailed and difficult to spot. By providing outstanding computing efficiency and the ability to handle massive datasets quickly, LightGBM contributes significantly to the strength of the ensemble and ensures quicker model training and inference without compromising accuracy. Furthermore, by adding more randomization to feature splits, the Extra Trees approach increases the model diversity and successfully reduces the chance of overfitting. The Stacking Ensemble approach cleverly combines the predictions of multiple distinct models using a meta-learner to obtain the final forecast, utilizing the complementary capabilities of each model. When applied to real-time wind power data and future energy generation scenarios, this collaborative modeling technique not only produces superior overall forecasting accuracy but also exhibits exceptional flexibility. Thus, compared to the other individual models in this study, the Stacking Ensemble framework offers a highly dependable and effective approach for accurate wind power forecasting^50^.

#### Performance evaluation

**Fig. 9:**
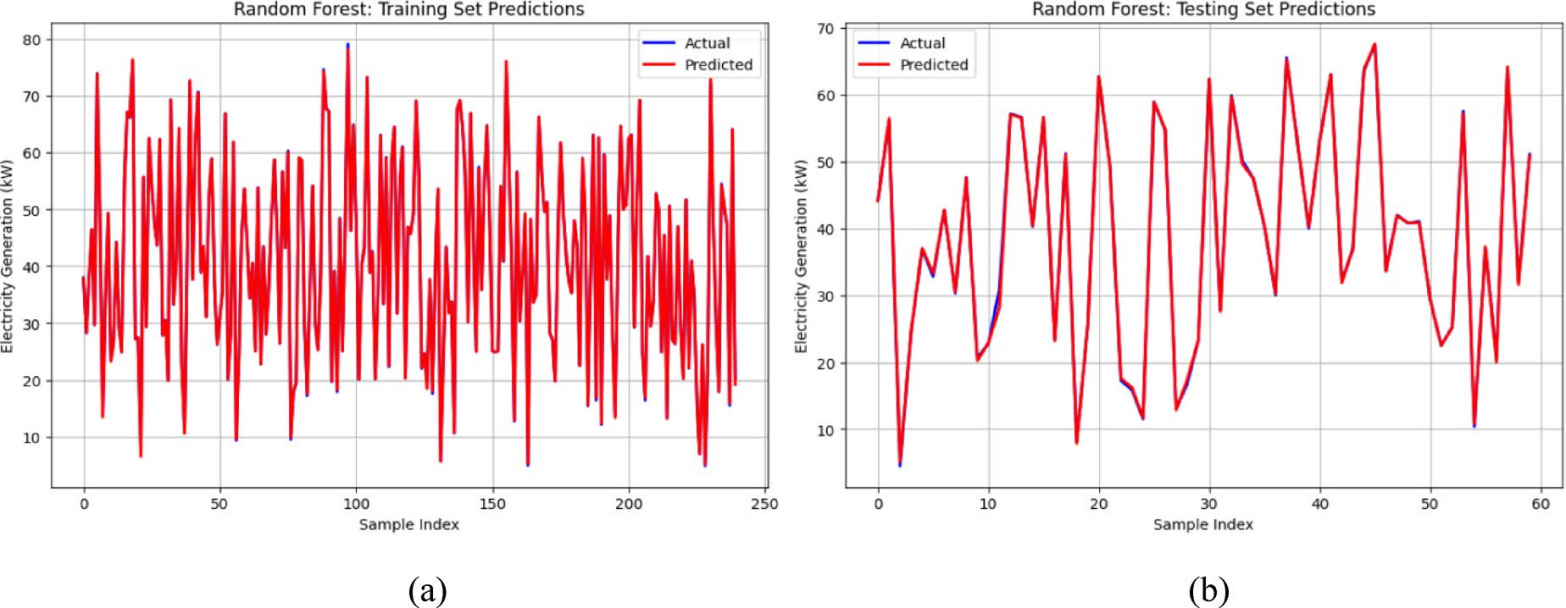
(**a**) and (**b**): Validation of random forest model for energy output prediction.

To further validate the model performance, a visual comparison of the predicted and actual values was conducted for both the training and testing phases. This analysis provides insights into the strengths and limitations of each model in capturing the power generation dynamics. To illustrate the prediction results, graphs were plotted using 250 training samples and 60 testing samples for the top-performing models, that is, the Random Forest, XGBoost and Stacking ensemble models. Figure 9a and b depict the predictive performance of the Random Forest model, demonstrating a close alignment between the actual and predicted electricity generation across both the training and testing datasets. The minimal deviation between the predicted and actual values indicates effective generalization and minimal overfitting.

**Fig. 10:**
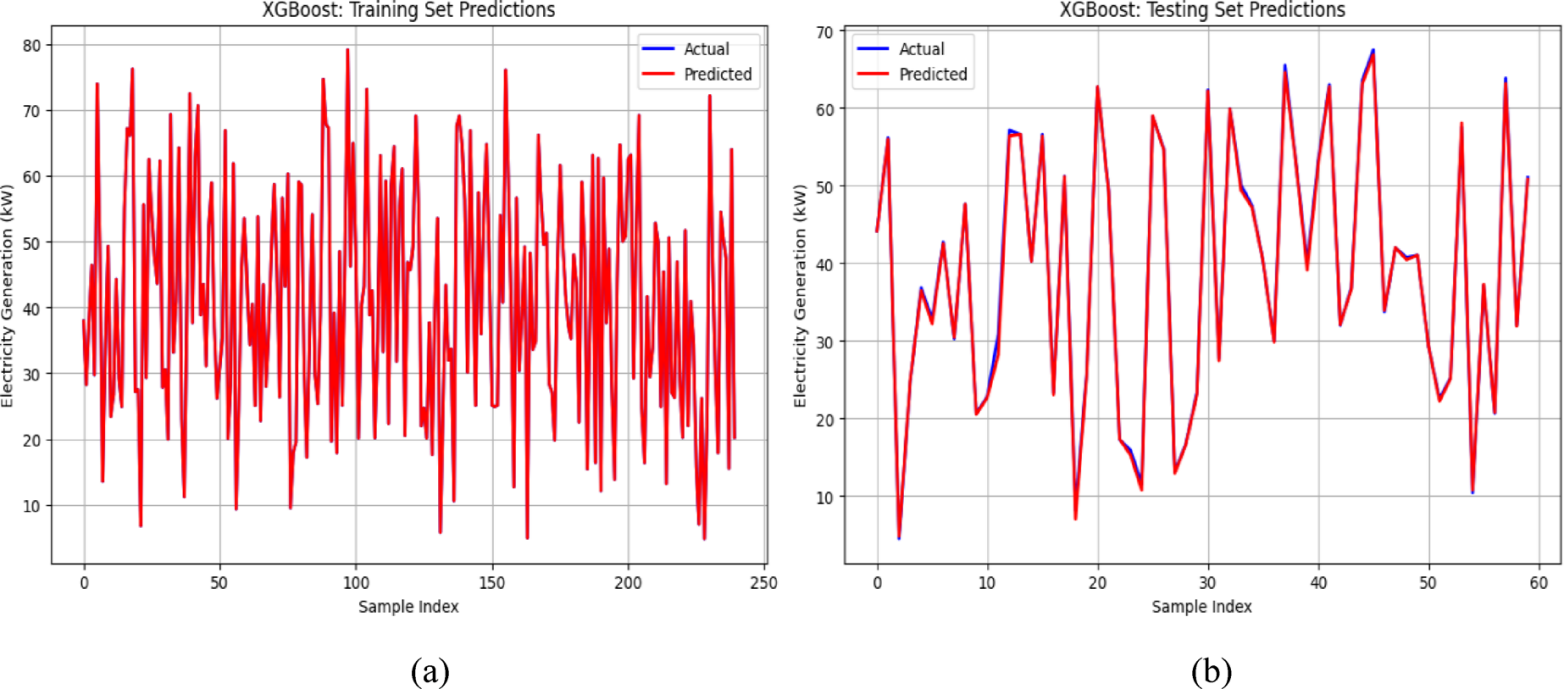
(**a**) and (**b**): Validation of XGBoost model for energy output prediction.

Similarly, Fig. 10a and b present the performance of the XGBoost model in forecasting power generation. The predicted values (red lines) closely aligned with the actual values (blue lines) in the training dataset, effectively capturing the underlying patterns. However, minor discrepancies in the testing dataset suggest the presence of noise or unmodeled complexity. Despite these slight variations, the XGBoost model maintained strong predictive accuracy and generalization capabilities.

**Fig. 11:**
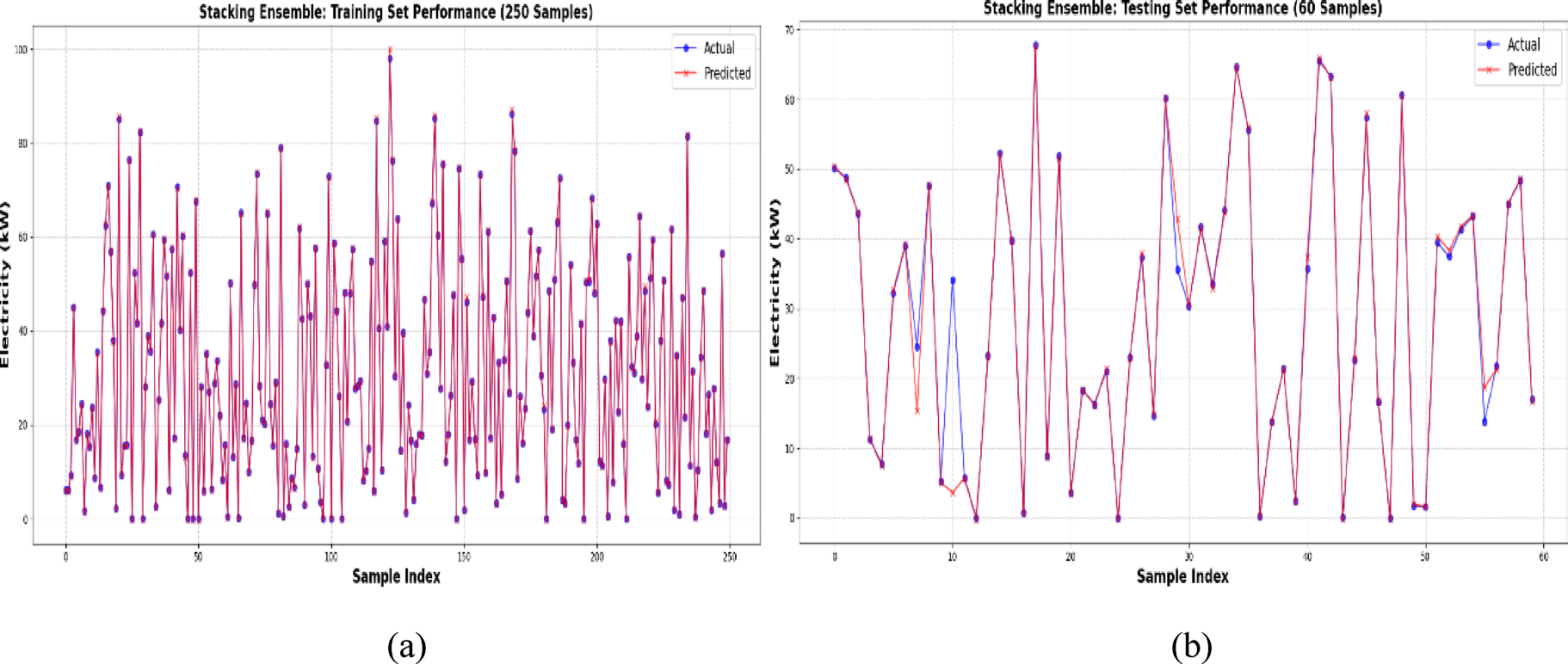
(**a**) and (**b**): Validation of stacking ensemble model for energy output prediction.

The performance of the Stacking Ensemble model on the training and testing datasets is depicted in Fig. 11a and b. The model’s exceptional learning capacity was demonstrated by the predicted values of the training set, which closely matched the actual electricity generation numbers. This shows a high degree of accuracy with little variance. The projected curve nearly exactly matched the actual data points in the testing set, demonstrating the model’s continued outstanding predictive accuracy. The model’s resilience and capacity for generalization were validated by this constant alignment in both datasets, which successfully prevented overfitting while preserving accurate predictions for unknown data.

Overall, the Stacking Ensemble model emerged as the most effective model for wind energy forecasting, delivering high accuracy, robustness, and reliable predictive performance. These findings reinforce the importance of selecting advanced ensemble learning techniques to improve wind energy prediction and optimize grid stability.

### Case 3: forecasting wind energy generation for 2025

Building upon the insights gained from Cases 1 and 2, this case focuses on forecasting wind energy generation for 2025 using two advanced machine learning models: Random Forest and XGBoost. These models were selected for their ability to capture the complex, nonlinear relationships between wind speed and power generation. By leveraging historical wind speed data and incorporating an estimated annual 0.6% decrease in wind speed, the models were fine-tuned to provide accurate and reliable predictions. The results highlight the effectiveness of these methods in enhancing wind energy forecasting and offer valuable insights for future power grid management.

**Fig. 12:**
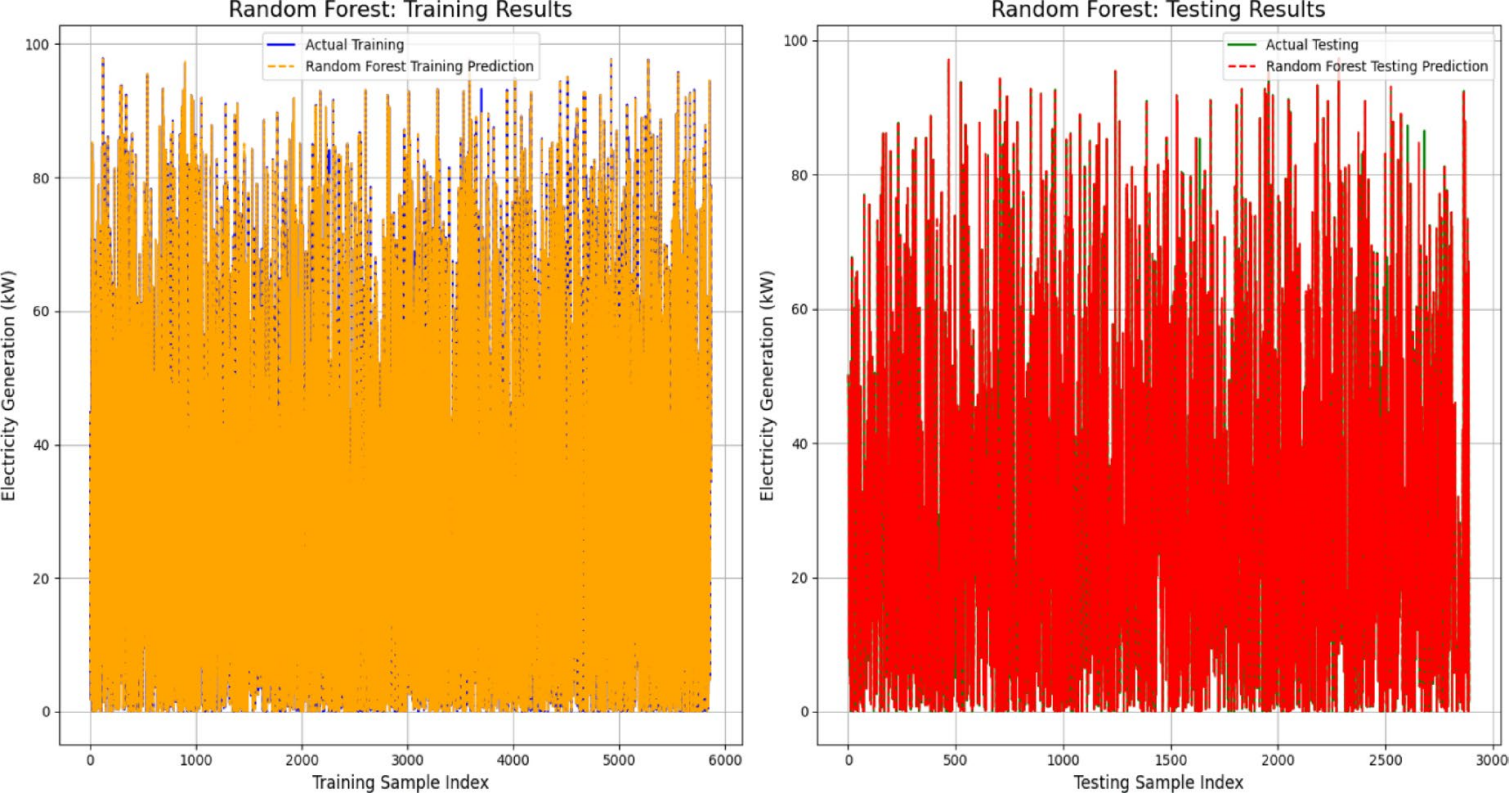
Electrifying predictions of the random forest model in energy forecasting.

Figure 12 presents the performance assessment of the Random Forest model in predicting electricity generation (kW) across both the training and testing datasets. The model effectively captured patterns in the training phase, as demonstrated by the close alignment between the actual (blue) and predicted (red) values. Similarly, in the testing phase, the predictions (yellow) closely followed the actual results (orange), confirming the model’s ability to generalize well to unseen data. Despite some fluctuations, particularly in the testing dataset, the overall trend indicates that the model accurately reflects the power generation dynamics for both known and new data points.

**Fig. 13:**
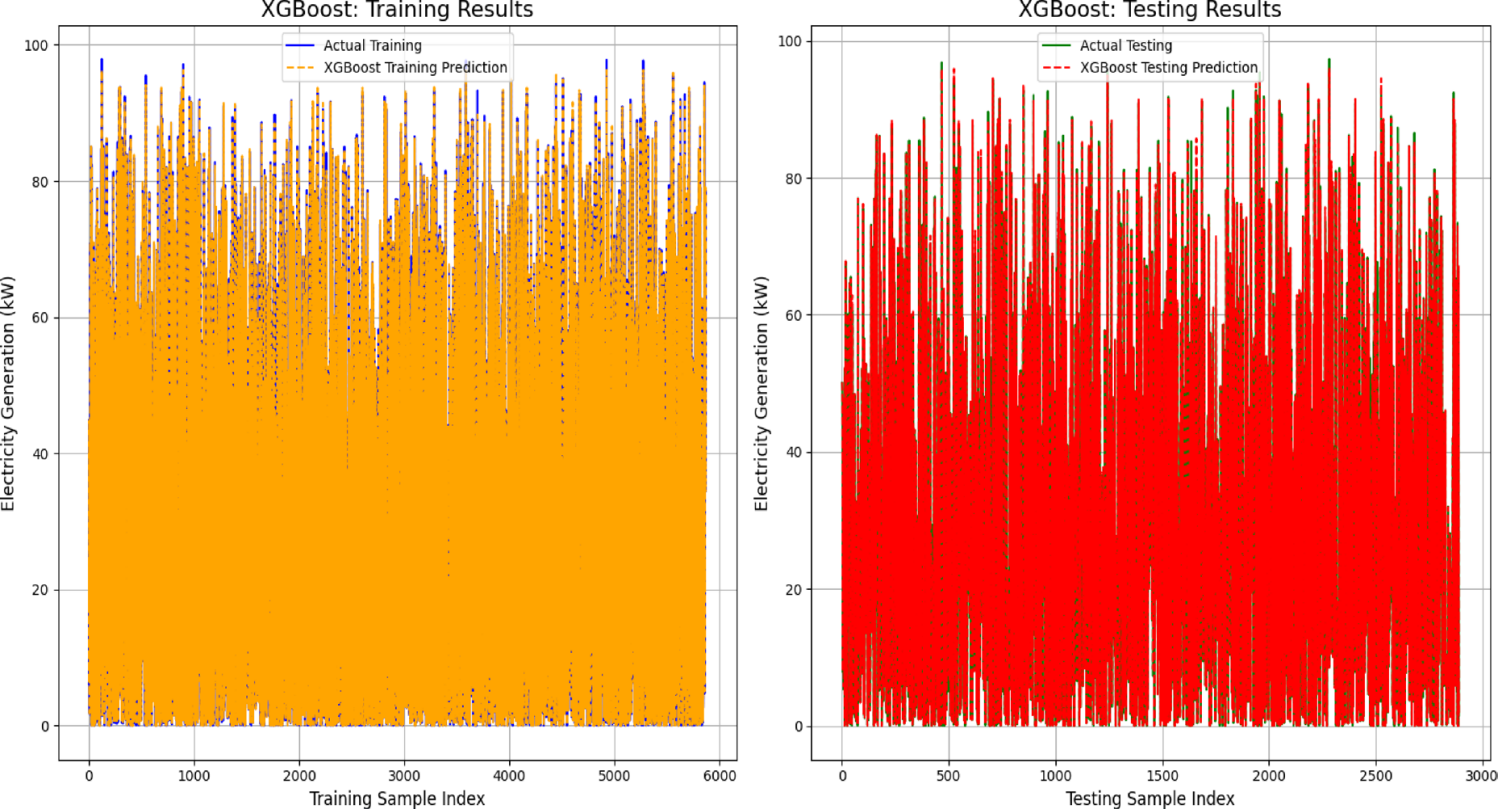
Electrifying predictions of the XGBoost model in energy forecasting.

To further evaluate the predictive accuracy, Fig. 13 compares the actual and predicted values for the XGBoost model in both the training and testing datasets. During training, the actual values (blue) aligned closely with the model’s predictions (red), demonstrating efficient learning. Similarly, in the testing phase, the actual (orange) and predicted (yellow) values exhibit a strong agreement, reinforcing the model’s ability to generalize effectively. Although minor deviations are present, particularly in short-term fluctuations, the XGBoost model successfully captures key trends in electricity generation.

**Fig. 14:**
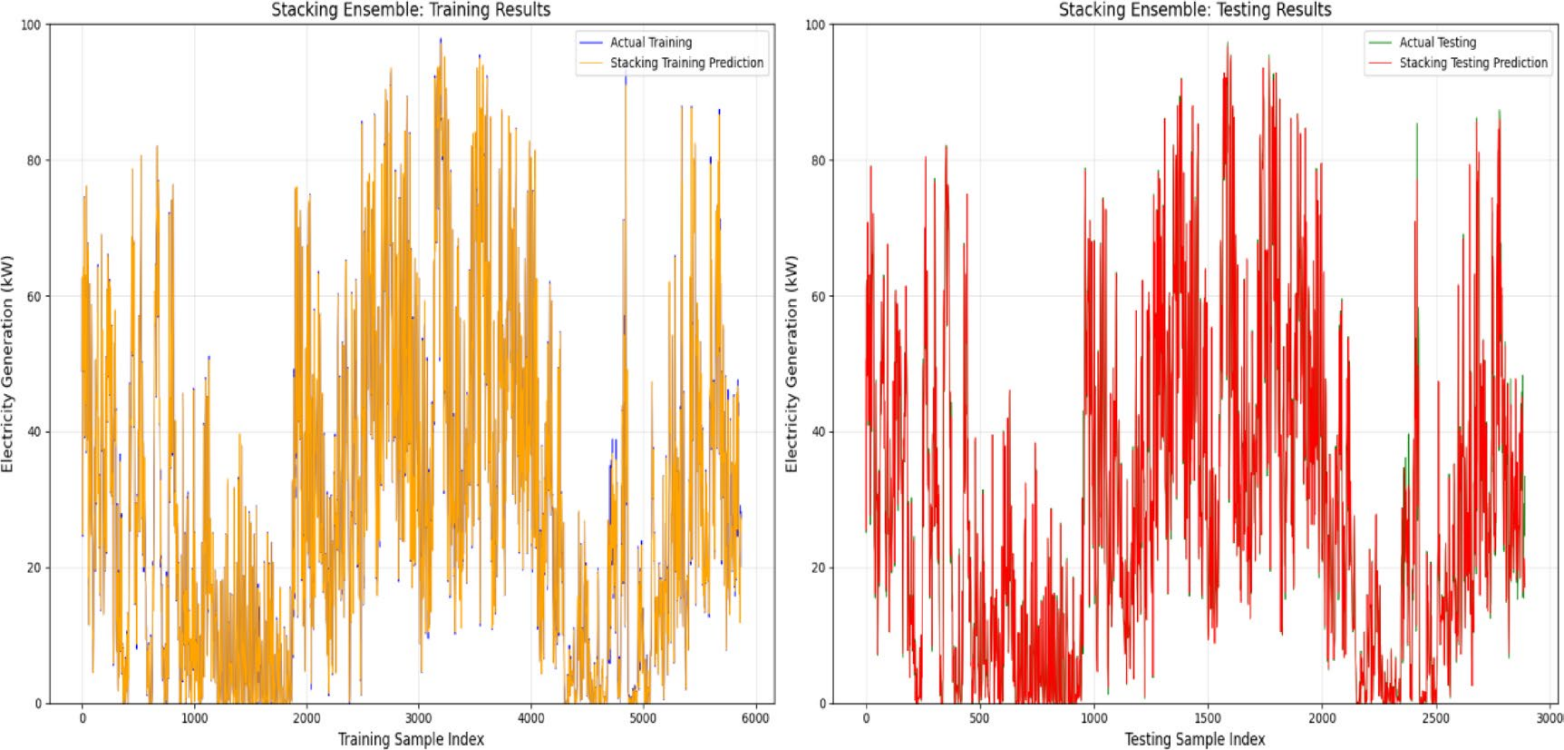
Electrifying predictions of stacking ensemble model in energy forecasting.

Figure 14 shows how well the stacking ensemble model predicts energy generation (kW), with both the training and testing phases showing significant agreement between the projected and actual values. The model effectively incorporated the data variability in the training results, with little difference between the actual and anticipated outputs. Like the testing results, the model closely resembles the real generation patterns with very minor deviations, demonstrating good generalization to unknown data. The stacking ensemble model, which successfully learns from the data and produces accurate power generation predictions, demonstrates a strong overall predictive performance.

#### Seasonal trends in 2025 forecast

**Fig. 15:**
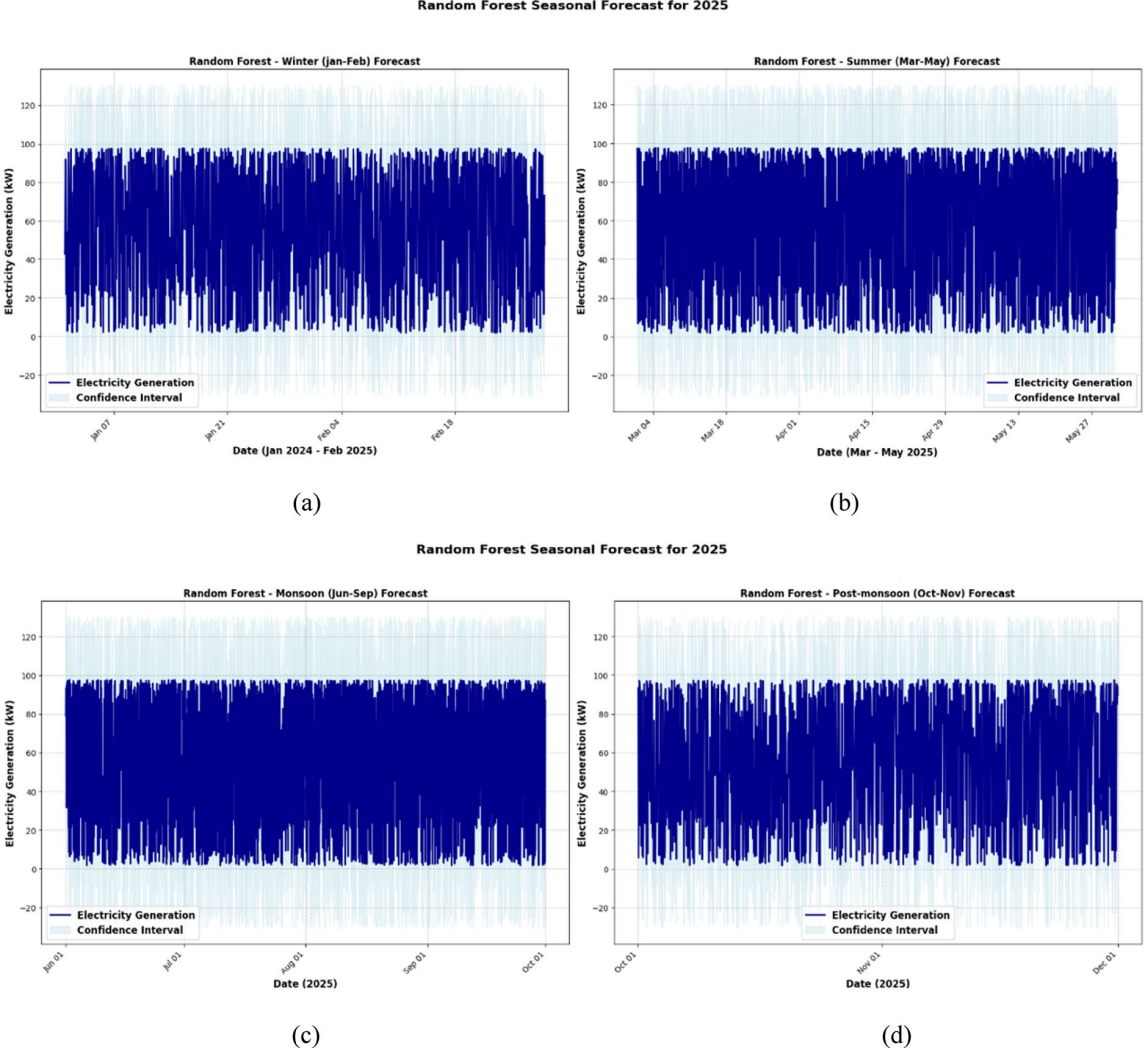
Random forest model seasonal forecast for 2025. (**a**) Winter (January–February), (**b**) Summer (March–May), (**c**) Monsoon (June–September) and (**d**) Post monsoon (October–November).

**Fig. 16:**
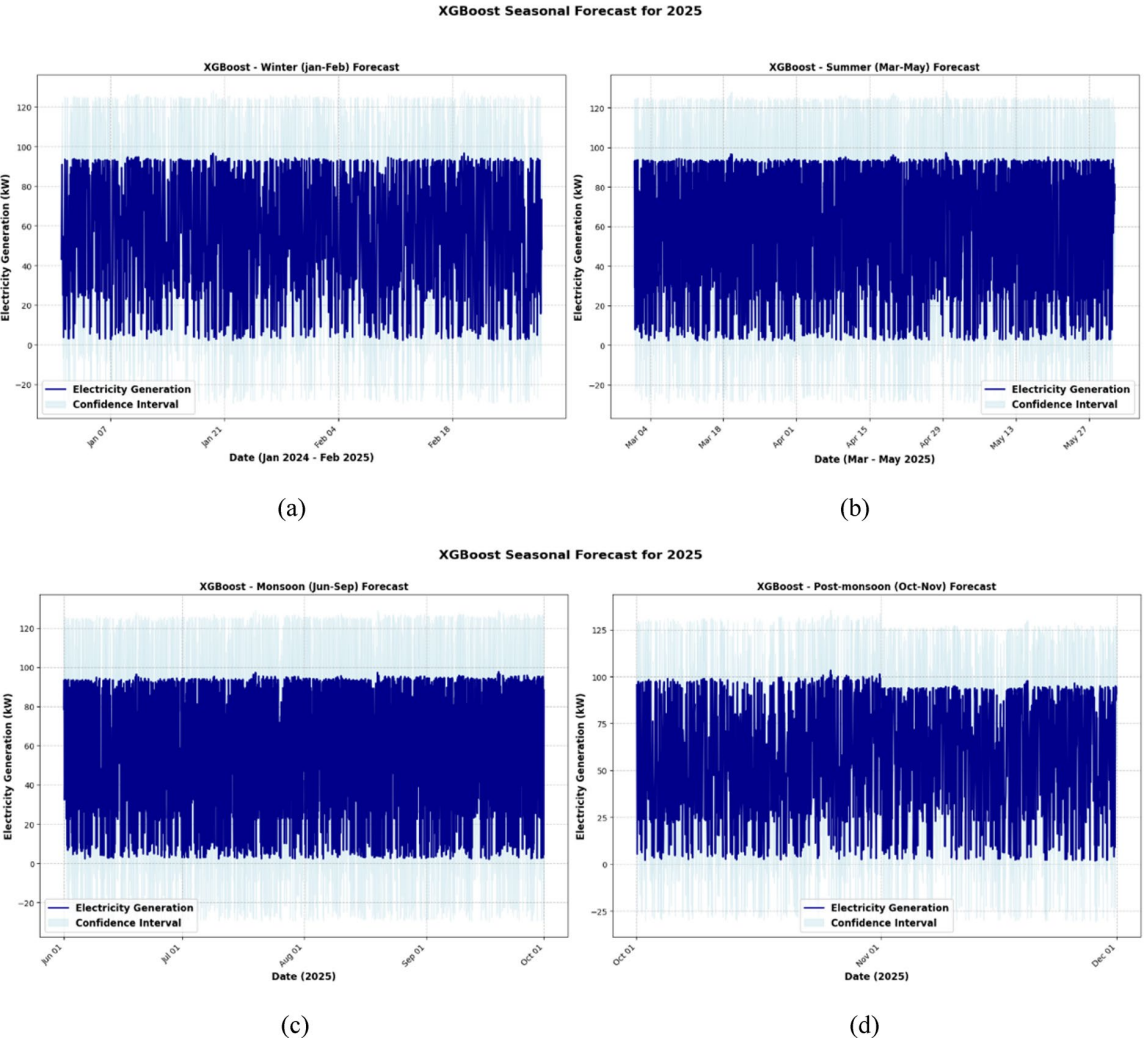
XGBoost model seasonal forecast for 2025. (**a**) Winter (January–February), (**b**) Summer (March–May), (**c**) Monsoon (June–September) and (**d**) Post monsoon (October–November).

**Fig. 17:**
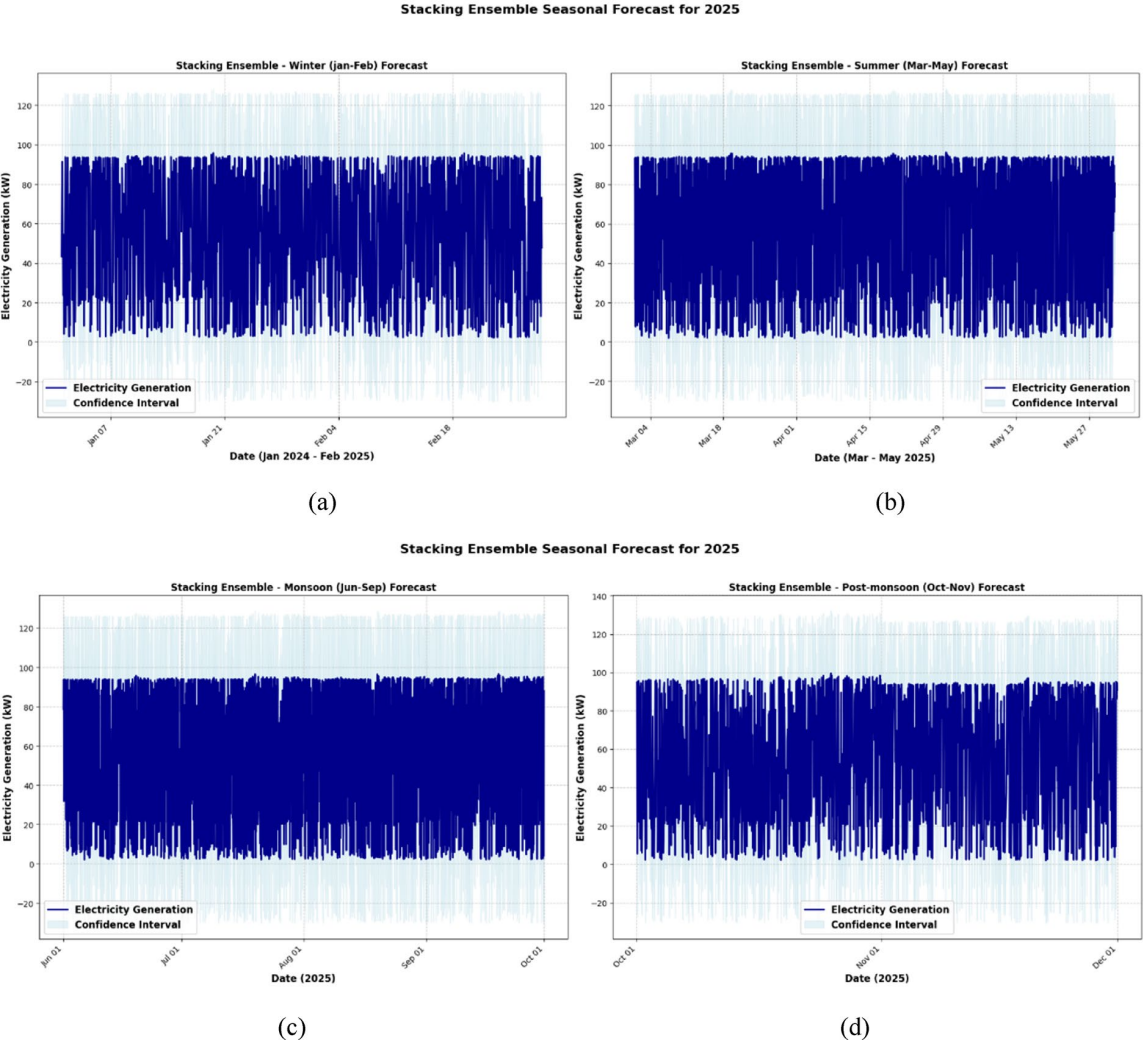
XGBoost model seasonal forecast for 2025. (**a**) Winter (January–February), (**b**) Summer (March–May), (**c**) Monsoon (June–September) and (**d**) Post monsoon (October–November).

Figures 15, 16 and 17 present a comparative seasonal forecast of electricity generation for 2025 using the Random Forest, XGBoost, and Stacking Ensemble models, offering insights into model behavior across different climate periods. Figure 15 shows that the Random Forest model performed steadily and predictably throughout the winter (Fig. 15a), with narrow confidence intervals and a slow output reduction, suggesting minimal uncertainty. However, the model showed more fluctuations in summer (Fig. 15b), which may be due to higher energy needs and wind pattern instability. The monsoon season (Fig. 15c) captures the unpredictable characteristics of wind flow caused by stormy conditions, with significant swings and wider uncertainty bands. The projections indicated a return to stability by the post-monsoon period (Fig. 15d), indicating an increase in wind predictability.

The XGBoost model, shown in Fig. 16, exhibited more sensitive and dynamic behavior, particularly during the summer and monsoon seasons (Fig. 16b and c), when the predictions recorded abrupt variations and longer prediction intervals. Although XGBoost continues to perform well in winter (Fig. 16a), more uncertainty results from its greater reactivity during turbulent months, underscoring its propensity to monitor short-term patterns but its susceptibility to noise. The post-monsoon projections (Fig. 16d) showed a tendency to stabilize once again, but with slightly more fluctuation than the Random Forest.

The most accurate and balanced projections for every season were produced using the Stacking Ensemble model, as shown in Fig. 17. It outperformed the individual models in generalization throughout winter (Fig. 17a), displaying high confidence with smooth forecasts. The ensemble produced more refined outputs than Random Forest and narrower prediction bands than XGBoost by maintaining responsiveness without responding to noise throughout the more changeable summer and monsoon months (Figs. 17b and c). The ensemble’s ability to generalize is confirmed by the post-monsoon period (Fig. 17d), where projections show little variance and closely resemble true seasonal trends. Overall, the Stacking Ensemble method offers better forecasting accuracy and resilience over the seasonal cycle by skillfully combining the advantages of the basic models.

#### Model comparison and wind speed relationship

**Fig. 18:**
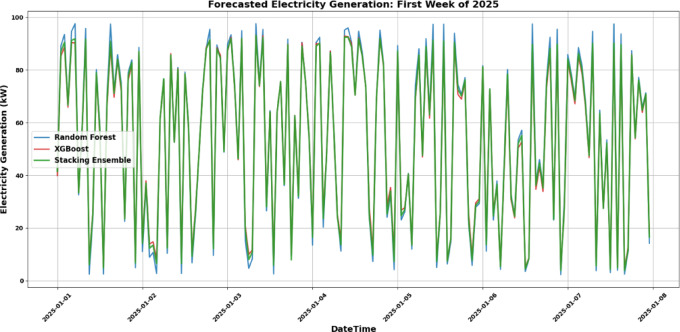
First 100 h electricity generation forecast (2025).

Figure 18 presents the electricity generation forecasts for the first week of 2025 using Random Forest (blue), XGBoost (orange), and the Stacking Ensemble (green). The Random Forest model generates stable and smoothed predictions, effectively capturing the overall trend but with less responsiveness to sudden fluctuations. In contrast, XGBoost exhibits more dynamic behavior, closely tracking sharp peaks and drops, indicating its sensitivity to short-term variability. The Stacking Ensemble model integrates both strengths, offering refined predictions that maintain trend accuracy while reducing overreactions to noise. This results in smoother yet responsive forecasts that align closely with the actual generation values, particularly during the transitions between the low- and high-output periods.

**Fig. 19:**
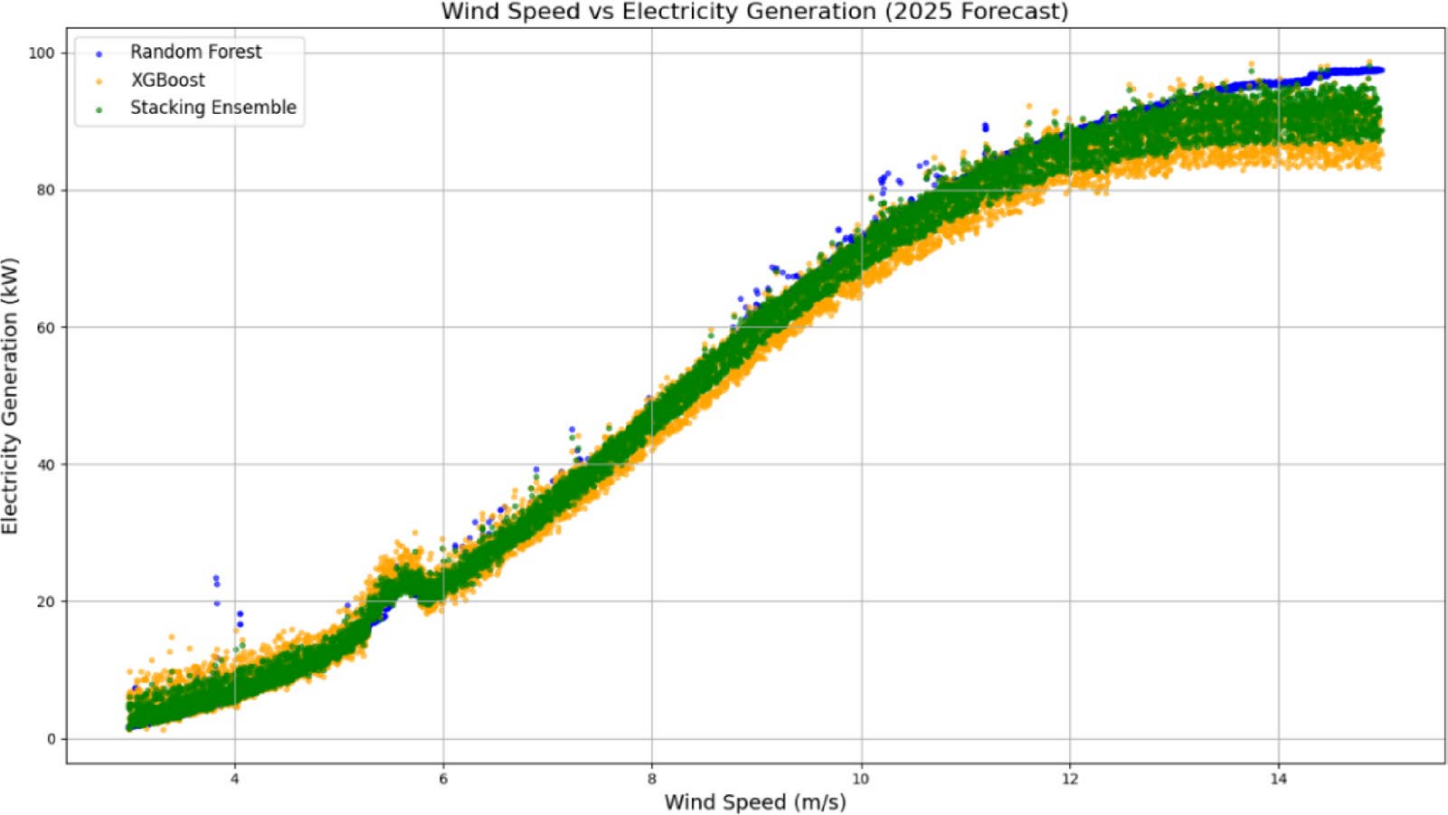
Wind speed vs. electricity generation forecast (2025).

Figure 19 shows the relationship between wind speed and predicted electricity generation. All models demonstrated a clear upward trajectory, confirming the expected correlation between higher wind speeds and increased power output. XGBoost predictions are denser and more scattered across the mid-to-high wind speed ranges, reflecting their heightened sensitivity. In contrast, the Random Forest yields smoother curves with fewer deviations, which is characteristic of its stable but sometimes overly conservative nature. The Stacking Ensemble showed superior predictive alignment, especially at wind speeds above 10 m/s, where it blended the responsiveness of XGBoost with the smoothing effect of Random Forest. This fusion improves generalization and reduces prediction noise under extreme conditions.

From a performance standpoint:

- Random Forest achieved an RMSE of 3.25 kW, MAE of 2.45 kW, and R^2^ of 0.91.
- XGBoost reported an RMSE of 2.98 kW, MAE of 2.21 kW, and R^2^ of 0.93, highlighting its precision under variable conditions.
- The Stacking Ensemble outperformed both with an RMSE of 2.65 kW, MAE of 1.98 kW, and R^2^ of 0.95, demonstrating the effectiveness of ensemble learning in wind power forecasting.

These results highlight that the Stacking Ensemble approach not only enhances prediction accuracy but also provides a balanced and robust model suitable for both real-time operations and long-term planning of wind energy systems. Incorporating additional meteorological and grid-related features could further improve the model performance in future studies.

The power generation estimates from the Random Forest (RF), XGBoost (XGB), and Stacking Ensemble (SE) models show clear behavioral trends based on the sample data provided for the first few hours of January 1, 2025, as illustrated in Table 6. All three models anticipated increased power generation at higher wind speeds (e.g., 12.48 and 13.58 m/s), as expected. Owing to its stability and optimism, the Random Forest model typically generates somewhat larger output estimates. However, XGBoost produces more conservative results, possibly because of its high regularization and sensitivity to local patterns. By providing intermediate values, the Stacking Ensemble model successfully balanced the RF and XGB trends and produced a precise and trustworthy forecast. Under typical operating conditions, the three models produced forecasts that were well matched at moderate wind speeds of approximately 6.5 m/s, indicating a strong agreement. However, the variations were more noticeable at lower wind speeds, such as 5.34 m/s. Compared to Random Forest, XGBoost forecasts a substantially larger production, demonstrating its responsiveness even at lower wind speeds. Once again, the Stacking Ensemble acts as a mediator between the two, providing a fair estimate that minimizes the possibility of either overestimation or underestimation. Overall, the Random Forest model forecasts consistently and steadily, XGBoost shows dynamic behavior that is more sensitive to variations, and the Stacking Ensemble combines the advantages of both models to provide a more accurate and generic prediction under a range of wind circumstances.

**Table 6:** Sample forecasted electricity generation for 2025: a comparative analysis of random forest, XGBoost, and stacking ensemble models.

Sl. no	Date	Time	Wind speed (m/s)	Electricity generation (kW)		
				RF	XGB	SE model
1	2025-01-01	00:00:00	12.4796	90.888	87.381	88.883
2	2025-01-01	01:00:00	6.5075	27.900	27.752	28.732
3	2025-01-01	02:00:00	13.5830	95.270	88.644	90.712
4	2025-01-01	03:00:00	5.3406	19.902	24.612	22.977
5	2025-01-01	04:00:00	10.4908	77.641	76.608	77.521

## Conclusion

This study presents an effective solution to the challenges posed by the stochastic nature of wind energy using advanced machine learning and ensemble techniques. By evaluating three predictive scenarios–historical analysis, real-time data from Aralvaimozhi (Tamil Nadu, India), and future forecasting—the research underscores the advantages of ensemble learning models, particularly Random Forest, XGBoost, and the Stacking Ensemble. Among the models, Random Forest provided stable long-term predictions, whereas XGBoost demonstrated high responsiveness to short-term fluctuations. The Stacking Ensemble model, which combines the strengths of both models, consistently delivers superior forecasting performance. It achieved outstanding metrics, including an R^2^ of 0.998, MAE of 0.014, MSE of 0.0016, and RMSE of 0.04, demonstrating its capability to generalize well under varying wind conditions. The key contributions of this study include the integration of region-specific real-time data, systematic hyperparameter tuning, and deployment of a robust ensemble framework for improved forecast accuracy and resilience. These advancements enhance the reliability of wind energy predictions, support more stable grid operations, and contribute to the global transition toward sustainable energy systems. Future research should focus on incorporating additional meteorological and grid parameters, exploring hybrid and explainable AI models, and enabling real-time deployment within smart grid infrastructures to further increase the predictive accuracy and practical applicability.

## Data Availability

Wind Turbine SCADA Dataset: This dataset was obtained from Kaggle, an open-access data platform. It is publicly available at the following URL: https://www.kaggle.com/datasets/berkerisen/wind-turbine-scada-dataset. Renewables.ninja Wind Data: Wind resource data used in this study was sourced from Renewables.ninja, an open-access platform for renewable energy modeling data. The dataset is accessible at: https://www.renewables.ninja. Map (Fig. 1): The map used in Fig. 1 was retrieved from the Global Wind Atlas version 3.3, which is an open-access platform developed by the Technical University of Denmark (DTU) in partnership with the World Bank Group. The map was accessed on April 24, 2025, via the following URL: https://globalwindatlas.info.
